# Synthesis of a novel chemotype via sequential metal-catalyzed cycloisomerizations

**DOI:** 10.3762/bjoc.8.153

**Published:** 2012-08-20

**Authors:** Bo Leng, Stephanie Chichetti, Shun Su, Aaron B Beeler, John A Porco

**Affiliations:** 1 Department of Chemistry and Center for Chemical Methodology and Library Development (CMLD-BU), Boston University, 590 Commonwealth Avenue, Boston, Massachusetts 02215, USA

**Keywords:** chemical diversity, cycloisomerization, cyclopropane, diyne, isochromene, π-acid

## Abstract

Sequential cycloisomerizations of diynyl *o*-benzaldehyde substrates to access novel polycyclic cyclopropanes are reported. The reaction sequence involves initial Cu(I)-mediated cycloisomerization/nucleophilic addition to an isochromene followed by diastereoselective Pt(II)-catalyzed enyne cycloisomerization.

## Introduction

**Scheme 1:**
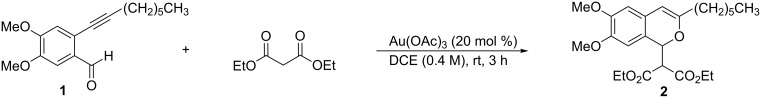
Cycloisomerization/nucleophilic addition of alkynyl benzaldehyde **1** to isochromene **2**.

Our laboratory has an ongoing interest in discovering transformations that afford novel chemotypes [1–4]. To this end, we have developed a reaction screening paradigm that enables the discovery of new reaction processes and chemotypes [5]. For example, we have conducted multidimensional reaction screens using alkynyl *o*-benzaldehyde scaffolds, which revealed a number of reactions affording novel polycyclic scaffolds, including Au(III)-catalyzed addition of diethyl malonate to **1** to afford isochromene **2** (Scheme 1). The chemotypes discovered in initial pilot studies have been further developed into library scaffolds and identified as biologically interesting structures [6]. Herein, we report the expanded utility of alkynyl *o*-benzaldehydes through a sequential metal-catalyzed cycloisomerization process to afford a novel polycyclic cyclopropane chemotype.

## Results and Discussion

**Figure 1:**
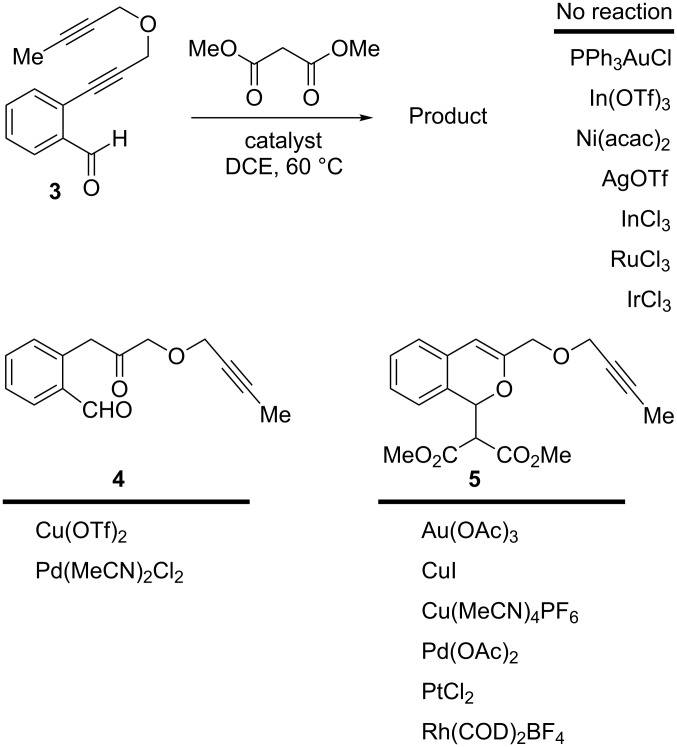
Reaction screen with diynyl benzaldehyde **3**.

**Scheme 2:**
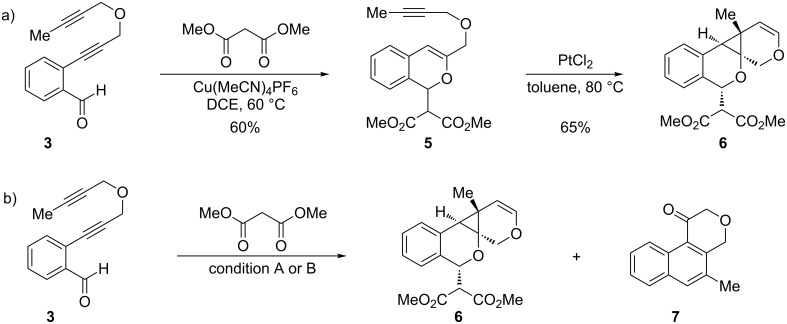
Sequential cycloisomerizations of substrate **3**. Condition A: PtCl_2_ (10 mol %), Cu(MeCN)_4_PF_6_ (10 mol %), toluene, 80 °C, 8 h (40%). Condition B: Step 1: Cu(MeCN)_4_PF_6_ (10 mol %), rt, 1 h. Step 2: PtCl_2_ (10 mol %), 80 °C, toluene, 5 h (89%).

In an effort to further explore the utility of alkynyl *o*-benzaldehydes as scaffolds for reaction screening, we designed a focused reaction screen with diynyl benzaldehyde [7] substrate **3**. Based on the cycloisomerization/addition reactions previously studied (Scheme 1), it was not clear at the outset of our study whether an *o*-alkynyl benzaldehyde containing an additional alkynyl moiety (**3**) would react to form an isochromene derivative or whether additional polycyclization would occur [8]. Accordingly, a reaction screen was conducted, evaluating a number of metal catalysts in the presence of diethyl malonate. From this focused reaction screen we identified three types of reactivity: (1) no reaction; (2) alkyne hydration (**4**); and (3) cycloisomerization leading to isochromene (**5**) (Figure 1). Many catalysts resulted in no reaction, including ones that might have been expected to catalyze cycloisomerization, such as AgOTf. Two catalysts, Cu(OTf)_2_ and Pd(MeCN)_2_Cl_2_, afforded only hydration of the alkyne. Interestingly, hydration was regioselective, which is possibly due to direction from the ether oxygen. We were most interested in metal catalysts that effected cycloisomerization of **3** to alkynyl isochromene **5**, which is an interesting enyne substrate with potential for further reactivity [9–10]. In the reaction screen of alkynyl benzaldehyde substrate **3**, we found that in the absence of optimization Cu(MeCN)_4_PF_6_ [11–13] afforded the highest isolated yield of **5** (60%) (Scheme 2).

**Figure 2:**
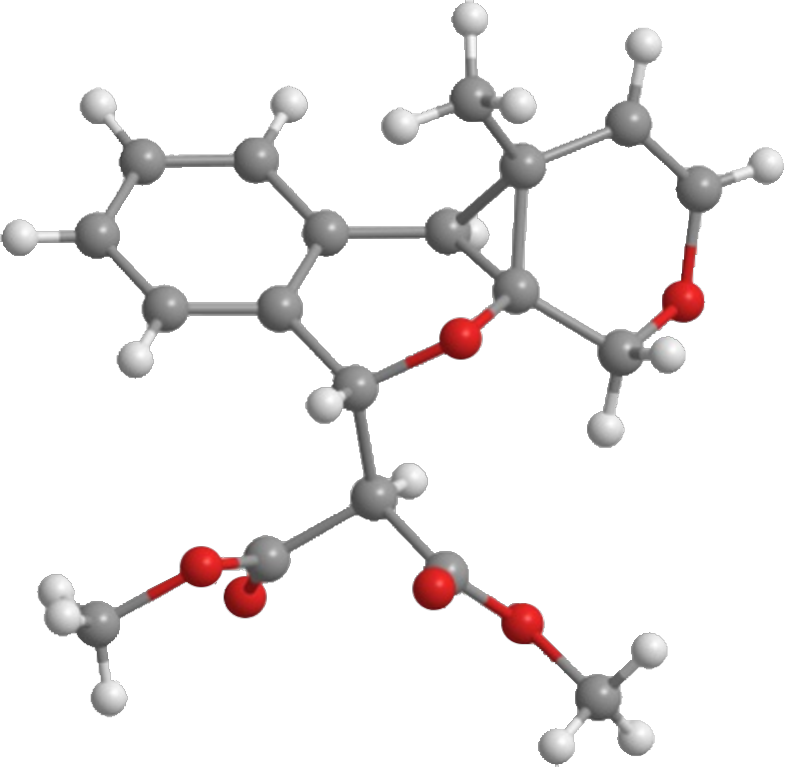
X-ray crystal structure of cyclopropane **6**.

As the production of isochromene **5** offered a unique opportunity for additional cycloisomerization processes, we elected to explore this manifold of reactivity. Based on reports by Echavarren and co-workers [14–15], we treated enyne **5** with PtCl_2_ at 80 °C in toluene [16–17]. The reaction afforded polycyclic cyclopropane **6** in good yield (65%) as a single diastereomer (Scheme 2a). Interestingly, reaction of **3** in the presence of only PtCl_2_ afforded exclusively isochromene **5** in low yield. Further studies revealed that a multicatalytic reaction system [18] utilizing both Cu(I) and Pt(II) [19] catalysts afforded the desired cyclopropane **6** in moderate yield (40%) along with ketone **7** (45%), derived from [4 + 2] cycloaddition of the benzopyrylium intermediate with the pendent alkyne [20] (Scheme 2b). However, better yields were observed when the initial cycloisomerization was carried out in the presence of Cu(MeCN)_4_PF_6_ followed by the addition of PtCl_2_ to the reaction mixture (Scheme 2b). Optimization of the one-pot conditions afforded exclusively **6** in good yield (89%). X-ray crystal analysis confirmed the structure and relative stereochemistry of polycyclic cyclopropane **6** (Figure 2, Supporting Information File 1).

We next focused on an evaluation of the general scope of the reaction with regard to aryl and alkyne substitution. Reaction utilizing an electron-poor trifluoromethyl-substituted diynyl benzaldehyde **8** was successful, producing product **9** in moderate yield (Table 1, entry 1). *m*-Methyl- and naphthyl-containing substrates **10** and **12** afforded polycyclic cyclopropanes **11** and **13** in 48 and 51% yields, respectively (Table 1, entries 2 and 3).

**Table 1:** Sequential cycloisomerizations of diynyl benzaldehyde substrates.

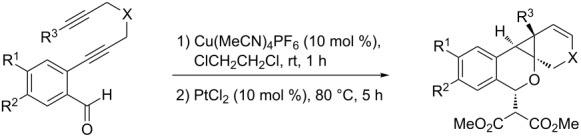							

entry	aldehyde	product	yield	entry	aldehyde	product	yield

1	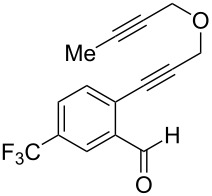 **8**	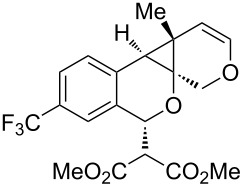 **9**	53%	5	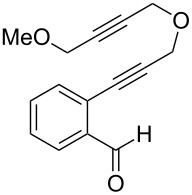 **16**	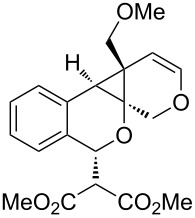 **17**	62%
2	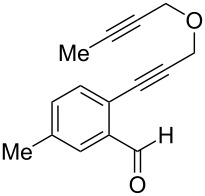 **10**	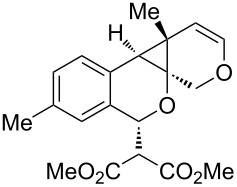 **11**	48%	6	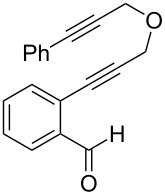 **18**	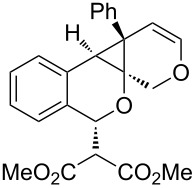 **19**	59%
3	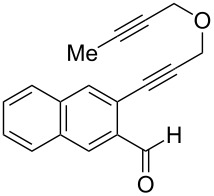 **12**	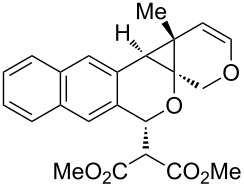 **13**	51%	7	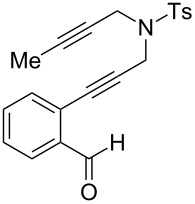 **20**	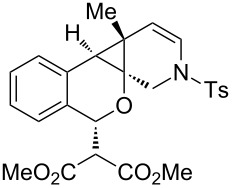 **21**	82%
4	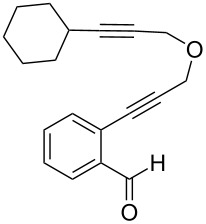 **14**	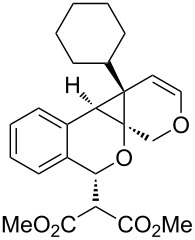 **15**	60%	8	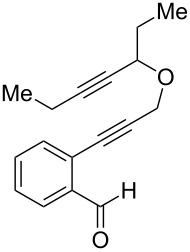 **22**	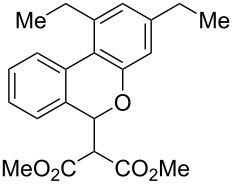 **23**	65%

We next explored substitution of the pendant alkyne. Reaction with cyclohexane diyne **14** afforded the fused cyclopropane **15** in moderate yield (60%), while methyl ether **16** afforded cyclopropane **17** in 62% yield. Phenyl substitution (**18**) also resulted in a moderate yield (59%, Table 1, entry 6). Substituting the oxygen with *N*-tosyl (**20**) afforded *N*-tosyl cyclopropane **21** in good yield (82%). Substitution at the internal methylene (**22**) resulted in a diverted reaction pathway (vida infra) affording product **23** exclusively in moderate yield (65%).

**Scheme 3:**
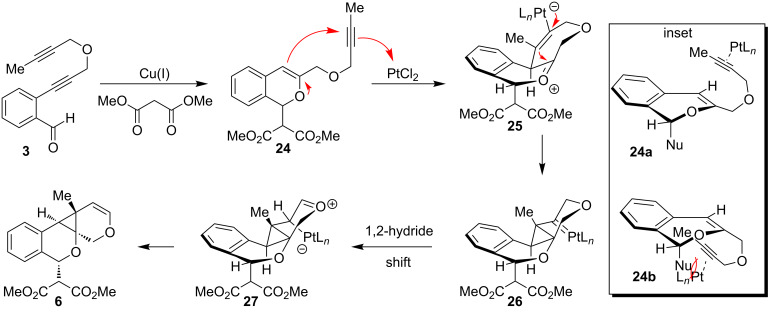
Proposed reaction pathway for diastereoselective, sequential cycloisomerization.

A proposed mechanistic pathway for diastereoselective, sequential cycloisomerizations is shown in Scheme 3. We propose the initial cycloisomerization and nucleophilic addition of diynyl benzaldehyde **3** and dimethyl malonate is catalyzed by Cu(I) to afford isochromene **24** [20–22]. Pt(II) π-coordination of the pendant alkyne of **24** followed by cyclization of the enol ether affords the seven-membered-ring metal-“ate” intermediate **25**. The cyclization occurs at the face opposite the malonate substituent (Nu, **24a**) to minimize steric interactions relative to **24b**, leading to the observed diastereoselectivity (Scheme 3, inset) [23–24]. Subsequent cyclopropane formation through addition of the vinyl metal to the oxonium intermediate affords metallocarbenoid **26**, which may then undergo a 1,2-hydride shift to intermediate **27** followed by elimination of the metal catalyst [25] to afford the observed cyclopropane product **6**.

**Scheme 4:**
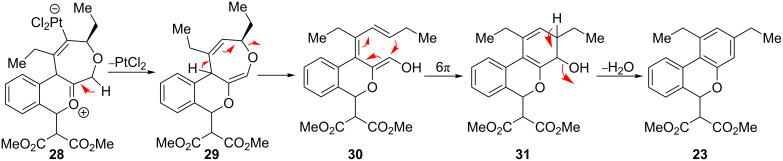
Proposed alternative reaction pathway affording **23**.

An alternative reaction pathway may be invoked for the ethyl-substituted substrate **22** leading to product **23** (Scheme 4). After initial cyclization of the enol ether with the Pt-activated alkyne, the resulting metal-“ate” intermediate **28** may undergo preferential elimination and proto-demetallation to afford 1,5-diene **29**. A second elimination results in the ring-opened triene **30**. Subsequent 6π-electrocyclization affords alcohol **31**, which aromatizes through loss of water to afford the observed isochromane **23**.

## Conclusion

We have described sequential cycloisomerizations of diynyl *o*-benzaldehyde substrates to access novel polycyclic cyclopropanes. The reaction sequence involves initial Cu(I)-mediated cycloisomerization/nucleophilic addition to an isochromene followed by diastereoselective Pt(II)-catalyzed enyne cycloisomerization. The chemistry reported herein illustrates the power of sequential cycloisomerization processes to provide access to novel chemotypes and chemical diversity from readily accessible building blocks [26]. Further transformations of the novel polycyclic cyclopropanes as well as additional studies employing reaction screening for metal-mediated processes is ongoing and will be reported in future publications.

## Experimental

**General Information:** All nuclear magnetic resonance spectra were recorded on either a Varian or Bruker spectrometer. ^1^H NMR spectra were recorded at 400 MHz at ambient temperature with CDCl_3_ as solvent, unless otherwise stated. ^13^C NMR spectra were recorded at 100.0 MHz at ambient temperature with CDCl_3_ as solvent, unless otherwise stated. Chemical shifts are reported in parts per million relative to CDCl_3_ (^1^H, δ 7.27; ^13^C, δ 77.0) and acetone-*d*_6_ (^1^H, δ 2.05; ^13^C, δ 30.8). Data for ^1^H NMR are reported as follows: chemical shift, multiplicity (ovrlp = overlapping, s = singlet, d = doublet, t = triplet, q = quartet, qt = quintuplet, m = multiplet), coupling constant in hertz, and integration. All ^13^C NMR spectra were recorded with complete proton decoupling. Analytical LC was performed on a 2.1 × 50 mm, 1.7 μM C18 column. Analytical thin-layer chromatography was performed by using 0.25 mm silica gel 60-F plates. Otherwise, flash chromatography was performed by using 200–400 mesh silica gel. Yields refer to chromatographically and spectroscopically pure materials, unless otherwise stated. Acetonitrile, CH_2_Cl_2_, THF, and toluene were purified by passing through two packed columns of neutral alumina. All reactions were performed under an argon atmosphere in oven-dried or flame-dried glassware.

**General procedure for the synthesis of alkynyl *o*-benzaldehydes: 2-(3-(but-2-ynyloxy)prop-1-ynyl)benzaldehyde.** To a solution of 2-bromobenzaldehyde (2.0 g, 10.8 mmol) and 1-(prop-2-ynyloxy)but-2-yne (1.4 g, 13 mmol) in Et_3_N (68 mL), was added tetrakis(triphenylphosphine)palladium(0) (0.38 g, 0.32 mmol). The reaction mixture was stirred at room temperature for 5 min. Copper(I) iodide (0.075 g, 0.4 mmol) was added, and the mixture was heated to 60 °C overnight. The mixture was concentrated in vacuo and purified by flash chromatography (SiO_2_, petroleum ether/EtOAc 4:1) to afford diynyl benzaldehyde **3** (1.5 g, 7.1 mmol, 66%) as a viscous yellow oil. ^1^H NMR (400 MHz, CDCl_3_) δ 10.22 (s, 1H), 7.91 (d, *J* = 7.6 Hz, 1H), 7.57 (m, 2H), 7.46 (m, 1H), 4.54 (s, 2H), 4.29 (q, *J* = 2.4 Hz, 2H), 1.89 (t, *J* = 2.4 Hz, 3 H); ^13^C NMR (100 MHz, CDCl_3_) δ 191.6, 136.2, 133.8, 133.6, 129.0, 127.3, 126.1, 91.9, 83.7, 82.2, 74.2, 57.6, 57.1, 3.7; IR (thin film) ν_max_: 2920, 2852, 1697, 1594, 1477, 1450, 1350, 1274, 1244, 1193, 1138, 1076, 765 cm^−1^.

**General one-pot procedure for sequential cycloisomerization:** To a flame-dried round-bottom flask was added **3** (10 mg, 0.046 mmol), dimethyl malonate (5.8 μL, 0.05 mmol) and toluene (1.0 mL). To the reaction mixture was added tetrakis(acetonitrile)copper(I) hexafluorophosphate (1.7 mg, 0.005 mmol), and the reaction mixture was stirred at room temperature for 1 h. Platinum(II) chloride (1.2 mg, 0.005 mmol) was added and the reaction mixture was heated to 80 °C for 5 h. The reaction mixture was concentrated in vacuo and purified by flash chromatography (SiO_2_, petroleum ether/EtOAc 9:1 to 4:1) to afford the desired cycloisomerization product **6** (14 mg, 0.041 mmol, 89%) as a white solid. ^1^H NMR (400 MHz, CDCl_3_) δ 7.25 (m, 2H), 7.08 (m, 1H), 6.98 (d, *J* = 4.2 Hz, 1H), 6.11 (d, *J* = 5.6 Hz, 1H), 5.28 (d, *J* = 10.4 Hz, 1H), 5.07 (d, *J* = 5.6 Hz, 1H), 4.33 (d, *J* = 10.0 Hz, 1H), 3.92 (d, *J* = 10.8 Hz, 1H), 3.83 (s, 3H), 3.66 (d, *J* = 10.0 Hz, 1H), 3.49 (s, 3H), 2.51 (s, 1H), 0.73 (s, 3H); ^13^C NMR (100 MHz, CDCl_3_) δ 167.3, 166.4, 141.0, 135.8, 133.7, 130.8, 130.3, 128.9, 126.2, 111.1, 75.0, 63.9, 62.7, 59.4, 53.2, 52.7, 30.5, 26.4, 12.2; IR (thin film) ν_max_: 2953, 2926, 2870, 1761, 1741, 1679, 1639, 1493, 1435, 1341, 1253, 1194, 1144, 1073, 1018, 912, 774, 749 cm^−1^; HRMS–ESI^+^ (*m*/*z*): [M + Na]^+^ calcd for C_19_H_20_O_6_, 367.1158; found, 367.1189.

## Supporting Information

File 1 Characterization data, spectra, and crystal structure data.
