# Supplementary material for: Synthesis of a novel chemotype via sequential metal-catalyzed cycloisomerizations
Source: Beilstein J Org Chem. 2012 Aug 20;8:1338–43. doi: 10.3762/bjoc.8.153 (PMC3458758; doi:10.3762/bjoc.8.153)
Supplement: File 1 — Characterization data, spectra, and crystal structure data. [file Beilstein_J_Org_Chem-08-1338-s001.pdf]

Supporting Information

for

Synthesis of a novel chemotype via sequential metal-catalyzed cycloisomerizations

Bo Leng, Stephanie Chichetti, Shun Su, Aaron B. Beeler, and John A. Porco, Jr.*

Address: Department of Chemistry and Center for Chemical Methodology and Library Development (CMLD-BU), Boston University, 590 Commonwealth Avenue, Boston, Massachusetts 02215

E-mail: John A. Porco, Jr. - porco@bu.edu

* Corresponding author

Characterization data, spectra, and crystal structure data

Table of contents

General information S2

Characterization data S3

^{1}H and ^{13}C NMR spectra for selected compounds S8

X-ray crystal structure data S27

General information

^{1}H NMR spectra were recorded on a 400 MHz spectrometer at ambient temperature. ^{13}C NMR spectra were recorded on a 100 MHz spectrometer at ambient temperature with complete proton decoupling. Chemical shifts are reported in parts per million relative to the residue solvent peak ($CDCl_3$, ^{1}H: δ 7.27; ^{13}C δ 77.23), unless otherwise stated. Data for ^{1}H NMR are reported as follows: chemical shift, integration, multiplicity (app = apparent, br = broad, ovrlp = overlapping, s = singlet, d = doublet, t = triplet, q = quartet, m = multiplet) and coupling constants. Infrared spectra were recorded on a Nicolet Nexus 670 FT-IR spectrophotometer. High-resolution mass spectra were obtained in the Boston University Chemical Instrumentation Center by using a Waters Q-TOF spectrometer. Analytical thin layer chromatography was performed on 0.25 mm silica gel 60-F plates. Flash chromatography was performed by using 200–400 mesh silica gel (Scientific Absorbent Incorporated). All other reagents were used as supplied by Sigma-Aldrich, Lancaster, Fluka, Strem, and Bachem Chemicals. All reactions were performed in flame-dried glassware under an argon atmosphere unless otherwise noted.

Characterization data

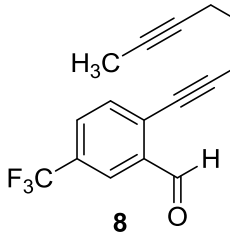

8

2-(3-(But-2-ynyloxy)prop-1-ynyl)-5-(trifuoromethyl)benzaldehyde. Prepared according to the general procedure reported for **3** and purified by flash chromatography (SiO_2, petroleum ether/EtOAc, 4:1) to afford **8** as a yellow oil (0.85 g, 3.0 mmol, 77%). ^{1}H NMR (400 MHz, $CDCl_3$) δ 1.87 (t, 3 H, J = 2.3 Hz), 4.28 (q, 2 H, J = 2.3 Hz), 4.54 (s, 2 H), 7.69 (d, 1 H, J = 8.1 Hz), 7.77 (d, 1 H, J = 8.1 Hz), 8.16 (s, 1 H), 10.51 (s, 1 H); ^{13}C NMR (100 MHz, $CDCl_3$) δ 190.0, 136.3, 134.1, 132.2, 131.1, 130.8, 130.0, 129.2, 124.4, 94.6, 83.8, 80.9, 73.9, 57.7, 56.8, 3.6; IR (thin film) ν_{max}: 2923, 2856, 1703, 1615, 1330, 1272, 1170, 1129, 1075, 916, 843, 723 cm^{-1}; HRMS–ESI$^+$ (m/z): $[M + H]^+$ calcd for $C_{15}H_{11}F_3O_2$, 281.0789; found, 281.0775.

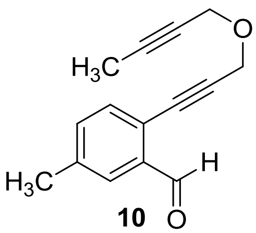

10

2-(3-(But-2-ynyloxy)prop-1-ynyl)-5-methylbenzaldehyde. Prepared according to the general procedure described for **3** and purified by flash chromatography (SiO_2, petroleum ether/EtOAc, 85:15) to afford **10** as a yellow solid (1.6 g, 7 mmol, 70%). ^{1}H NMR (400 MHz, $CDCl_3$) δ 1.86 (t, 3 H, J = 2.3 Hz), 2.37 (s, 3 H), 4.27 (q, 2 H, J = 2.3 Hz), 4.50 (s, 2 H), 7.21 (d, 1 H, J = 7.9 Hz), 7.35 (s, 1 H), 7.78 (d, 1 H, J = 7.9 Hz), 10.43 (s, 1 H); ^{13}C NMR (100 MHz, $CDCl_3$) δ 191.1, 144.8, 133.8, 129.8, 127.2, 125.9, 91.2, 83.5, 82.2, 74.1, 57.4, 56.9, 21.5, 3.6; IR (thin film) ν_{max}: 2920, 2851, 1694, 1599, 1442, 1349, 1256, 1137, 1113, 1076, 823, 798 cm^{-1}; HRMS–ESI$^+$(m/z): $[M + Na]^+$ calcd for $C_{15}H_{14}O_2$, 249.0891; found, 249.0900.

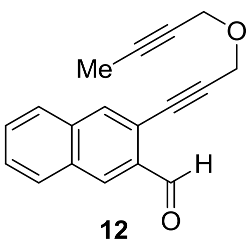

12

3-(3-(But-2-ynyloxy)prop-1-ynyl)-2-naphthaldehyde: Prepared according to the general procedure reported for **3** and purified by flash chromatography (SiO_2, petroleum ether/EtOAc, 95:5) to afford **12** as an orange oil (1.9 g, 7 mmol, 85%). ^{1}H NMR (400 MHz, $CDCl_3$) δ 10.76 (s, 1H), 8.49 (t, 1 H, J = 5.2 Hz), 7.94 (d, 1 H, J = 8.4 Hz), 7.88 (m, 2 H), 7.67 (m, 2 H), 4.71 (s, 2 H), 4.38 (q, 2 H, J = 2.4 Hz), 1.91 (t, 3 H, J = 2.4 Hz); ^{13}C NMR (100 MHz, $CDCl_3$) δ 191.9, 135.6, 134.6, 133.1, 129.3, 129.1, 128.4, 127.7, 127.1, 126.5, 121.9, 97.8, 83.8, 80.0, 74.0, 57.7, 57.1, 3.6; IR (thin film) ν_{max}: 2850, 1690, 1591, 1432, 1235, 1138, 1072, 821, 766 cm^{-1}; HRMS–ESI$^+$(m/z): $[M + Na]^+$ calcd for $C_{18}H_{15}O_2$, 263.1072; found, 263.1087.

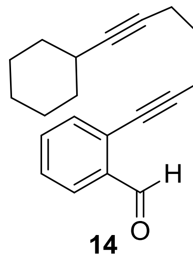

14

2-(3-(3-Cyclohexylprop-2-ynyloxy)prop-1-ynyl)benzaldehyde. Prepared according to the general procedure reported for **3** and purified by flash chromatography (SiO_2, petroleum ether/EtOAc, 9:1) to afford **14** as a yellow oil (0.85g, 3 mmol, 56%). ^{1}H NMR (400 MHz, $CDCl_3$) δ 10.51 (s, 1 H), 7.89 (d, 1 H, *J* = 7.8 Hz, 7.56 (m, 2 H), 7.44 (t, 1 H, *J* = 7.8 Hz), 4.53 (s, 2 H), 4.32 (d, 2 H, *J* = 2.0 Hz), 2.41 (t, 1 H, *J* = 9.0 Hz), 1.79 (m, 2 H), 1.68 (m, 2 H), 1.45 (m, 3 H), 1.28 (m, 3 H); ^{13}C NMR (100 MHz, $CDCl_3$) δ 191.4, 136.0, 133.7, 133.5, 128.8, 127.2, 126.0, 92.2, 91.9, 82.0, 74.6, 57.5, 56.8, 32.5, 29.0, 25.8, 24.8; IR (thin film) ν_{max}: 3462, 3079, 2983, 1641, 1396, 1022, 923, 856, 821, 764, 698, 668 cm^{-1}; HRMS–ESI$^+$ (*m*/*z*): $[M + Na]^+$ calcd for $C_{19}H_{20}O_2$, 303.1361; found, 303.1334.

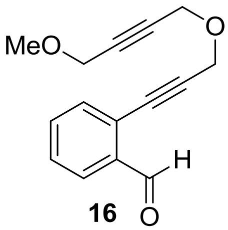

16

2-(3-(4-Methoxybut-2-ynyloxy)prop-1-ynyl)benzaldehyde. Prepared according to the general procedure reported for **3** and purified by flash chromatography (SiO_2, petroleum ether/EtOAc, 9:1) to afford **16** as a yellow oil (0.68 g, 2.8 mmol, 52%). ^{1}H NMR (400 MHz, $CDCl_3$) δ 10.51 (s, 1 H), 7.92 (d, 1 H, *J* = 7.2 Hz), 7.58 (m, 2 H), 7.46 (m, 1 H), 4.55 (s, 2 H), 4.39 (s, 2 H), 4.16 (s, 2 H), 3.40 (s, 3 H); ^{13}C NMR (100 MHz, $CDCl_3$) δ 191.3, 136.0, 133.7, 133.5, 128.9, 127.3, 125.7, 91.4, 83.1, 82.3, 81.3, 59.8, 57.6, 57.1, 57.1; IR (thin film) ν_{max}: 2933, 2897, 2851, 1729, 1695, 1601, 1451, 1352, 1189, 1095, 945, 760 cm^{-1}; HRMS–ESI$^+$ (*m*/*z*): $[M + H]^+$ calcd for $C_{14}H_{12}O_2$, 243.1021; found, 243.1039.

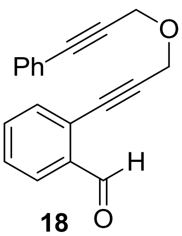

18

2-(3-(3-Phenylprop-2-ynyloxy)prop-1-ynyl)benzaldehyde. Prepared according to the general procedure reported for **3** and purified by flash chromatography (SiO_2, petroleum ether/EtOAc, 95:5) to afford **18** as an orange oily compound (1.1 g, 4 mmol, 76%). ^{1}H NMR (400 MHz, $CDCl_3$) δ 10.53 (s, 1 H); 7.90 (d, 1 H, *J* = 7.2 Hz), 7.56 (m, 2 H), 7.45 (m, 3 H), 7.32 (m, 3 H), 4.61 (s, 2 H), 4.56 (s, 2 H); ^{13}C NMR (100 MHz, $CDCl_3$) δ 191.3, 136.0, 133.7, 133.5, 131.8, 128.9, 129.0, 128.3, 127.2, 125.8, 122.2, 91.6, 87.1, 83.9, 82.3, 57.7, 57.2; IR (thin film) ν_{max}: 3061, 2848, 1697, 1594, 1490, 1443, 1352, 1193, 1078, 758, 692 cm^{-1}; HRMS–ESI$^+$ (*m*/*z*): $[M + Na]^+$calcd for $C_{19}H_{14}O_2$, 297.0891; found, 297.0895.

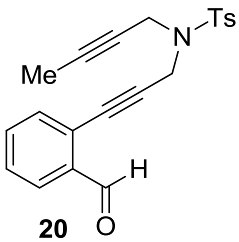

20

***N*-(But-2-ynyl)-*N*-(3-(2-formylphenyl)prop-2-ynyl)-4-methylbenzene sulfonamide.** Prepared according to the general procedure reported for **3** and purified by flash chromatography (SiO_2, petroleum ether/EtOAc, 4:1) to afford **20** as a yellow oil (1.0 g, 2.7 mmol, 52%). ^{1}H NMR (400 MHz, $CDCl_3$) δ 10.06 (s, 1 H), 7.85 (dd, 1 H, J_1 = 6.5 Hz, J_2 = 1.3 Hz), 7.73 (d, 2 H, *J* =8.3 Hz), 7.51 (dt, 1 H, J_1 = 7.6 Hz, J_2 = 6.1 Hz), 7.42 (t, 1 H, *J* = 7.7 Hz), 7.33 (d, 1 H, *J* = 7.7 Hz), 7.22 (d, 2 H, *J* = 7.2 Hz), 4.47 (s, 2 H), 4.16 (q, 2 H, *J* = 2.3 Hz), 2.96 (s, 1 H), 2.88 (s, 1 H), 2.31 (s, 3 H), 1.71 (t, 3 H, *J* = 2.3 Hz); ^{13}C NMR (100 MHz, $CDCl_3$): δ 191.2, 144.2, 136.2, 135.5, 133.8, 133.6, 129.7, 129.1, 128.1, 127.3,

126.0, 89.3, 82.5, 81.6, 71.6, 37.6, 37.4, 21.6; IR (thin film) ν_{max} :2921, 2849, 1696, 1596, 1595, 1477, 1449, 1351, 1193, 1163, 1093, 901, 816, 765, 730, 660, 570 cm^{-1}; HRMS–ESI$^+$ (*m*/*z*): $[M + H]^+$ calcd for $C_{21}H_{19}O_3S$, 366.1164; found, 366.1162.

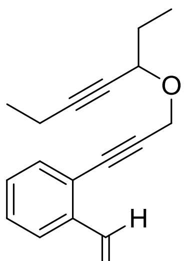

22

2-(3-(Hept-4-yn-3-yloxy)prop-1-ynyl)benzaldehyde. Prepared according to the general procedure reported for **3** and purified by flash chromatography (SiO_2, petroleum ether/EtOAc, 4:1) to afford **22** as a viscous orange oil (0.78 g, 3.0 mmol, 57%). ^{1}H NMR (400 MHz, $CDCl_3$) δ 10.52 (s, 1 H), 7.89 (d, 1 H, *J* = 7.3 Hz), 7.55 (m, 2 H), 7.43 (t, 1 H, *J* = 7.7 Hz), 4.54 (dd, 2 H, J_1 = 15.3 Hz, J_2 = 16.0 Hz), 4.25 (t, 1 H, *J* = 6.2 Hz), 1.15 (t, 3 H, *J* = 7.4 Hz), 1.02 (t, 3 H, *J* = 7.4 Hz); ^{13}C NMR (100 MHz, $CDCl_3$) δ 191.4, 136.0, 133.6, 133.4, 128.7, 127.0, 126.2, 92.6, 88.7, 81.4, 76.7, 70.2, 56.1, 28.9, 13.9, 12.3, 9.6; IR (thin film) ν_{max} : 3065, 2974, 2937, 2877, 2849, 2746, 2232, 1697, 1595, 1477, 1452, 1388, 1334, 1274, 1243, 1159, 1069, 962, 824, 764, 639 cm^{-1}; HRMS–ESI$^+$ (*m*/*z*): $[M + Na]^+$ calcd for $C_{17}H_{18}O_2$, 277.1204; found, 277.1227.

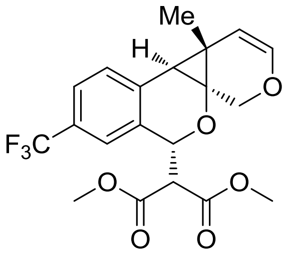

9

Cyclopropane isochromane 9. Prepared according to the general procedure reported for **6** and purified by flash chromatography (SiO_2, petroleum ether/EtOAc, 4:1) to afford **9** as a yellow oil (8.2 mg, 0.02 mmol, 53%). ^{1}H NMR (400 MHz, $CDCl_3$) δ 7.61 (d, 1 H, *J* = 7.61 Hz), 7.48 (d, 1 H, *J* = 7.9 Hz), 7.35 (s, 1 H), 5.45 (d, 1 H, *J* = 10.6 Hz), 5.19 (d, 1 H, *J* = 5.7 Hz), 4.44 (d, 1 H, *J* = 10.2 Hz), 4.01 (d, 1 H, *J* = 10.7 Hz), 3.96 (s, 3 H), 3.79 (d, 1 H, *J* = 10.2 Hz), 3.61 (s, 3 H), 2.68 (s, 1 H), 0.86 (s, 3 H); ^{13}C NMR (100 MHz, $CDCl_3$): δ 166.7, 165.8, 141.3, 135.2, 134.4, 130.5, 128.5, 125.4, 123.0 (d, *J* = 3 Hz), 110.5, 74.4, 63.8, 62.3, 59.0, 53.1, 52.6, 30.1, 30.0, 27.0, 12.0; IR (thin film) ν_{max}: 3062, 2957, 2927, 2873, 1742, 1639, 1622, 1436, 1410, 1330, 1258, 1192, 1126, 1075, 1020, 912, 743, 703 cm^{-1}; HRMS–ESI$^+$ (*m*/*z*): $[M + Na]^+$ calcd for $C_{20}H_{19}F_3O_6$, 435.1031; found, 435.1048.

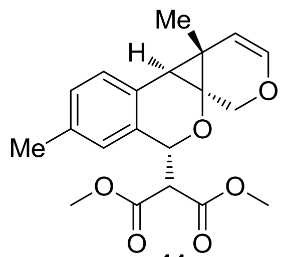

11

Cyclopropane isochromane 11. Prepared according to the general procedure reported for **6** and purified by flash chromatography (SiO_2, petroleum ether/EtOAc, 4:1) to afford **11** as a yellow oil (9.8 mg, 0.027 mmol, 48%). ^{1}H NMR (400 MHz, chloroform-*d*) δ ppm 7.02 (s, 1 H), 6.85 (d, *J* = 7.6 Hz, 1 H), 6.80 (d, *J* = 7.6 Hz, 1 H), 6.06 (d, *J* = 5.6 Hz, 1 H), 5.23 (d, *J* = 10.4 Hz, 1 H), 5.02 (d, *J* = 5.6 Hz, 1 H), 4.30 (d, *J* = 10.4 Hz, 1 H), 3.86 (d, *J* = 10.8 Hz, 1 H), 3.77 (s, 3 H), 3.62 (d, *J* = 10.4 Hz, 1 H), 3.45 (s, 3 H), 2.40 (s, 1 H), 2.24 (s, 3 H), 0.67 (s, 3 H); ^{13}C NMR (100 MHz, $CDCl_3$) δ ppm 167.1, 166.1, 140.7, 138.3, 130.6, 130.5, 130.3, 126.9, 125.7, 110.9, 74.7, 63.5, 62.5, 59.3, 52.9, 52.5, 30.3, 26.0, 21.2, 12.0; IR (thin film) ν_{max} : 2954, 2925, 2870, 1740, 1640, 1614, 1501, 1341, 1256, 1195, 1145, 1073, 1020, 917, 821, 738 cm^{-1}; HRMS–ESI$^+$ (*m*/*z*): $[M + Na]^+$ calcd for $C_{14}H_{12}O_2$, 381.1314; found, 381.1337.

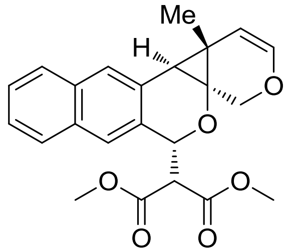

13

Cyclopropane isochromane 13. Prepared according to the general procedure reported for **6** and purified by flash chromatography (SiO_2, petroleum ether/EtOAc, 4:1) to afford **13** as a brown solid (9.2 mg, 0.023 mmol, 61%). ^{1}H NMR (400 MHz, $CDCl_3$) δ 7.92 (d, 1 H, 8.4 Hz), 7.75 (d, 1 H, *J* = 8.5 Hz), 7.57 (d, 1 H, *J* = 8.5 Hz), 7.47 (m, 2 H), 7.08 (d, 1 H, *J* = 8.5 Hz), 6.19 (d, 1 H, *J* = 5.7 Hz), 5.42 (d, 1 H, *J* = 10.6 Hz), 5.16 (d, 1 H, *J* = 5.7 Hz), 3.97 (d, 1 H, *J* = 10.6 Hz), 3.81 (s, 3 H), 3.72 (d, 1 H, *J* = 10.2 Hz), 3.43 (s, 3 H), 2.78 (s, 1 H), 0.62 (s, 3 H); ^{13}C NMR (100 MHz, $CDCl_3$): δ 169.1, 166.2, 141.3, 133.3, 132.7, 131.3, 128.5, 127.7, 126.7, 126.4, 123.9, 123.7, 110.6, 75.5, 64.4, 62.7, 59.2, 53.1, 52.6, 28.4, 26.8, 11.5; IR (thin film) ν_{max}: 3059, 2953, 2926, 2870, 1762, 1740, 1640, 1435, 1336, 1262, 1223, 1143, 1072, 1020, 972, 821, 744 cm^{-1}; HRMS–ESI$^+$ (*m*/*z*): [M + Na]$^+$ calcd for $C_{23}H_{22}O_6$, 417.1314; found, 417.1331.

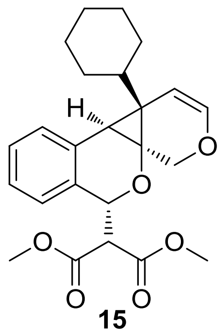

15

Cyclopropane isochromane 15. Prepared according to the general procedure reported for **6** and purified by flash chromatography (SiO_2, petroleum ether/EtOAc, 9:1) to afford **15** as a yellow oil (9.0 mg, 0.022 mmol, 60%). ^{1}H NMR (400 MHz, $CDCl_3$) δ 7.23 (d, 1 H, *J* = 8.8 Hz), 7.15 (t, 1 H, *J* = 7.4 Hz), 7.03 (t, 1 H, *J* = 7.6 Hz), 6.92 (d, 1 H, *J* = 7.7 Hz), 6.14 (d, 1 H, *J* = 5.8 Hz), 5.26 (d, 1 H, *J* = 10.5 Hz), 1.28 (m, 3 H), 6.15 (d, 1 H, *J* = 5.8 Hz), 4.27 (d, 1 H, *J* = 10.1 Hz), 3.89 (d, 1 H, *J* = 10.5 Hz), 3.79 (s, 3 H), 3.60 (d, 1 H, *J* = 10.1 Hz), 2.47 (s, 1 H), 3.43 (s, 3 H), 2.41 (t, 1 H, *J* = 9.0 Hz), 1.79 (m, 2 H), 1.68 (m, 2 H), 1.45 (m, 3 H); ^{13}C NMR (100 MHz, $CDCl_3$): δ 167.2, 166.5, 141.9, 133.7, 130.5, 130.2, 128.1, 125.9, 125.6, 106.2, 74.8, 64.0, 63.2, 59.2, 52.9, 52.5, 34.1, 33.7, 30.8, 30.5, 27.3, 26.3, 26.2; IR (thin film) ν_{max}: 2954, 2925, 2870, 1740, 1640, 1614, 1501, 1435, 1341, 1256, 1195, 1145, 1073, 1020, 821, 738 cm^{-1}; HRMS–ESI$^+$ (*m*/*z*): [M + Na]$^+$ calcd for $C_{24}H_{28}O_6$, 435.1784; found, 435.1800.

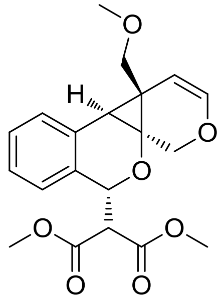

17

Cyclopropane isochromane 17. Prepared according to the general procedure reported for **6** and purified by flash chromatography (SiO_2, petroleum ether/EtOAc, 4:1) to afford **17** as a yellow oil (9.6 mg, 0.026 mmol, 62%). ^{1}H NMR (400 MHz, $CDCl_3$) δ 7.31 (d, 1 H, *J* = 7.6 Hz), 7.27 (m, 1 H), 7.13 (t, 1 H, *J* = 7.6 Hz), 7.02 (d, 1 H, *J* = 7.6 Hz), 6.24 (d, 1 H, *J* = 5.8 Hz), 5.35 (d, 1 H, *J* = 5.8 Hz), 5.34 (s, 1 H), 4.39 (d, 1 H, *J* = 10.3 Hz), 3.94 (d, 1 H, *J* = 10.5 Hz), 3.74 (d, 1 H, *J* = 10.1 Hz), 3.65 (s, 3 H), 3.50 (s, 3 H), 3.04 (dd, 2 H, J_1 = 10.8 Hz, J_1 = 17.0 Hz), 3.08 (s, 3 H), 2.70 (s, 1 H); ^{13}C NMR (100 MHz, $CDCl_3$): δ 166.9, 166.1, 142.0, 133.4, 130.3, 129.4, 128.6, 126.4, 125.9, 107.0, 74.8, 68.8, 64.3, 62.3, 59.0, 58.5, 53.0, 52.5, 30.2, 30.0; IR (thin film) ν_{max}: 2954, 2927, 1739, 1641, 1493, 1436, 1342, 1260, 1195, 1150, 1104, 1066, 1019, 914, 769, 737 cm^{-1}; HRMS–ESI$^+$ (*m*/*z*): [M + Na]$^+$ calcd for $C_{20}H_{22}O_7$, 397.1263; found, 397.1230.

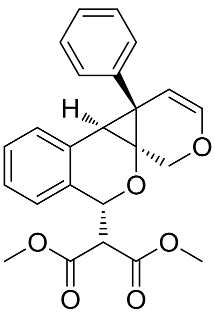

19

Cyclopropane isochromane 19. Prepared according to the general procedure reported for **6** and purified by flash chromatography (SiO_2, petroleum ether/EtOAc, 4:1) to afford **19** as a brown oil (9.5 mg, 0.023 mmol, 64%). ^1H NMR (400 MHz, $CDCl_3$) δ 7.42 (d, 1 H, J = 7.4 Hz), 7.18 (t, 1 H, J = 7.5 Hz), 7.04 (m, 5 H), 6.93 (t, 1 H, J = 7.5 Hz), 6.70 (d, 1 H, J = 7.2 Hz), 6.18 (d, 1 H, J = 5.7 Hz), 5.19 (d, 1 H, J = 5.6 Hz), 5.05 (d, 1 H, J = 10.1 Hz),2.98 (s, 1 H), 4.51 (d, 1 H, J = 10.3 Hz), 3.90 (d, 1 H, J = 6.2 Hz), 3.88 (d, 1 H, J = 6.0 Hz), 3.83 (s, 3 H), 3.77 (d, 1 H, J = 7.6 Hz), 3.46 (s, 3 H); ^{13}C NMR(100 MHz, $CDCl_3$): δ 166.7, 165.8, 140.3, 136.9, 132.8, 131.2, 130.5, 130.0, 128.0, 127.7, 126.1, 126.0, 125.6, 111.9, 74.0, 64.8, 63.2, 61.9, 61.6, 60.0, 35.5, 32.8, 14.2, 13.8; IR (thin film) ν_{max}: 3027, 2982, 2937, 2870, 1757, 1733, 1636, 1603, 1496, 1446, 1369, 1301, 1261, 1175, 1129, 1027, 929, 753, 701 cm^{-1}; HRMS–ESI$^+$ (m/z): $[M + Na]^+$ calcd for $C_{26}H_{26}O_6$, 457.1627; found, 457.1631.

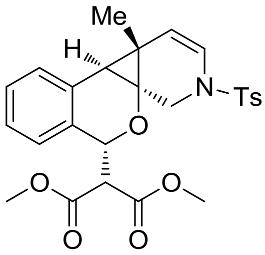

21

Cyclopropane isochromane 21. Prepared according to the general procedure reported for **6** and purified by flash chromatography (SiO_2, petroleum ether/EtOAc, 4:1) to afford **21** as a white solid (11.20 mg, 0.023 mmol, 82%). ^1H NMR (400 MHz, $CDCl_3$) δ 7.71 (d, 1 H, J = 8.1 Hz), 7.32 (d, 2 H, J = 8.1 Hz), 7.19 (t, 1 H, J = 7.5 Hz), 7.07 (t, 1 H, J = 7.5 Hz), 7.00 (d, 1 H, J = 7.5 Hz), 6.94 (d, 1 H, 7.6 Hz), 6.32 (d, 1 H, J =7.8 Hz), 5.26 (dd, 2 H, J_1 = 8.3 Hz, J_2 = 9.3 Hz), 4.28 (d, 1 H, J = 12.2 Hz), 3.92 (s, 3 H), 3.86 (d, 1 H, J = 11.2 Hz), 3.54 (s, 3 H), 2.99 (d, 1 H, J = 12.2 Hz), 2.35 (s, 3 H), 1.50 (s, 1 H), 0.64 (s, 3 H); ^{13}C NMR (100 MHz, $CDCl_3$): δ 166.8, 165.8, 144.2, 134.4, 133.1, 130.1, 129.7, 128.6, 126.9, 126.2, 126.0, 120.5, 117.8, 74.9, 65.6, 59.0, 53.0, 52.6, 42.7, 31.3, 27.0, 21.5, 11.8; IR (thin film) ν_{max}: 3062, 2954, 2926, 2877, 1763, 1633, 1597, 1493, 1435, 1398, 1346, 1272, 1170, 1107, 1018, 990, 915, 816, 737, 709, 677, 646 cm^{-1}; HRMS (m/z): $[M + H]^+$ calcd for $C_{26}H_{27}NO_7S$, 498.1586; found, 498.1571.

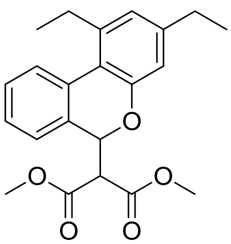

23

Dimethyl 2-(1,3-diethyl-6*H*-benzo[*c*]chromen-6-yl)malonate. Prepared according to the general procedure reported for **6** and purified by flash chromatography (SiO_2, petroleum ether/EtOAc, 4:1) to afford **23** as a yellow oil (9.8 mg, 0.026 mmol, 65%). ^1H NMR (400 MHz, chloroform-*d*) δ ppm 7.67 (d, J = 7.6 Hz, 1 H), 7.36 (t, J = 7.6 Hz, 1 H), 7.28 (d, J = 7.6 Hz, 1 H), 7.23 (m, 2 H), 6.84 (s, 1 H), 6.63 (s, 1 H), 5.76 (d, J = 10.8 Hz, 1 H), 3.86 (d, J = 10.4 Hz, 1 H), 3.78 (s, 3 H), 3.56 (s, 3 H), 3.00 (m, 2 H), 2.59 (q, J = 8 Hz, 2 H), 1.37 (t, J = 7.6 Hz, 3 H), 1.22 (t, J = 7.6 Hz, 3 H); ^{13}C NMR (100 MHz, chloroform-*d*) δ 166.9, 166.6, 152.4, 145.6, 141.3, 132.2, 129.7, 128.6, 126.8, 126.5, 126.3, 124.3, 119.3, 115.2, 75.5, 54.3, 52.7, 52.6, 28.6, 27.2, 15.6, 15.2; IR (thin film) ν_{max}: 2964, 2934, 2876, 1742, 1615, 1584, 1458, 1435, 1417, 1346, 1301, 1270, 1195, 1149, 1080, 1023, 983, 943, 864, 775, 742 cm^{-1}; HRMS–ESI$^+$ (m/z): $[M + Na]^+$ calcd for $C_{14}H_{12}O_2$, 391.1521; found, 391.1541.

^{1}H and ^{13}C NMR spectra for selected compounds

^{1}H and ^{13}C NMR of compound **3** ($CDCl_3$)

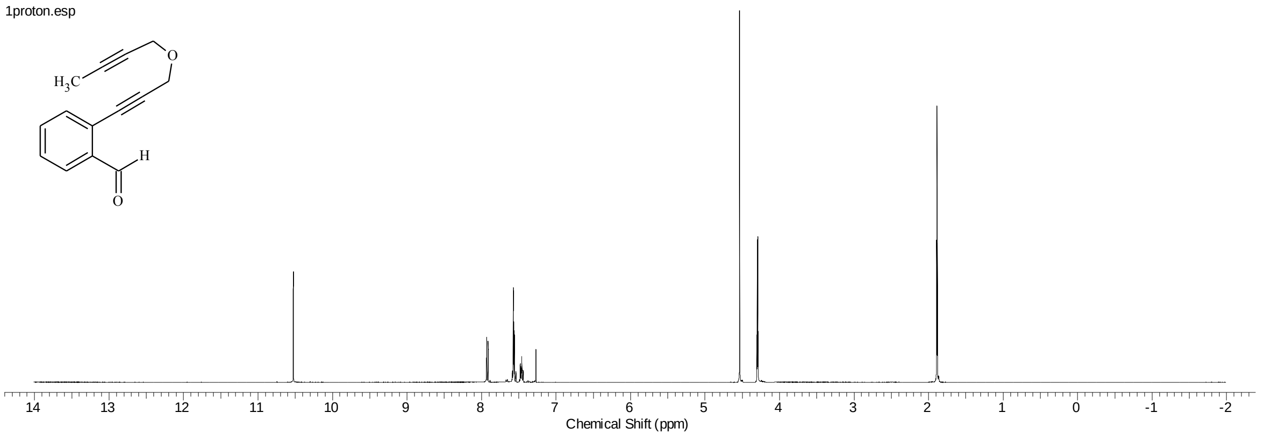

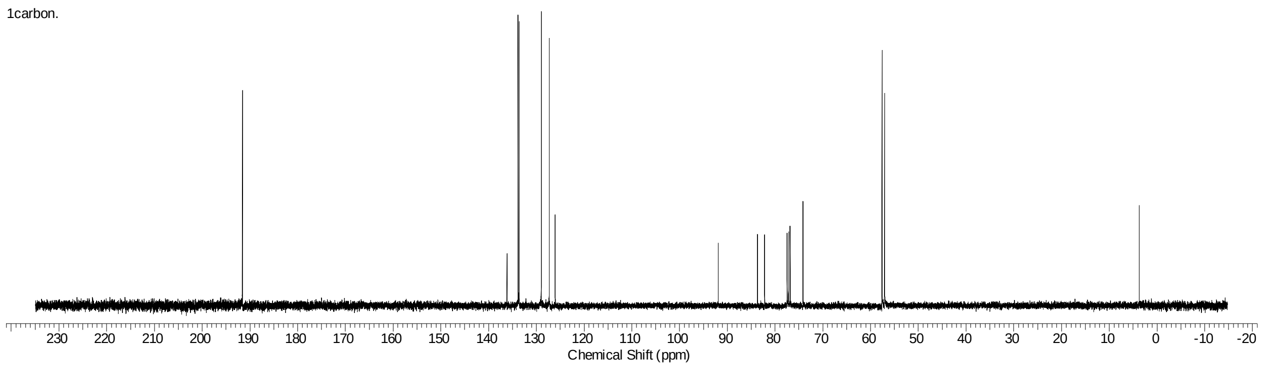

^{1}H and ^{13}C NMR of compound **8** ($CDCl_3$)

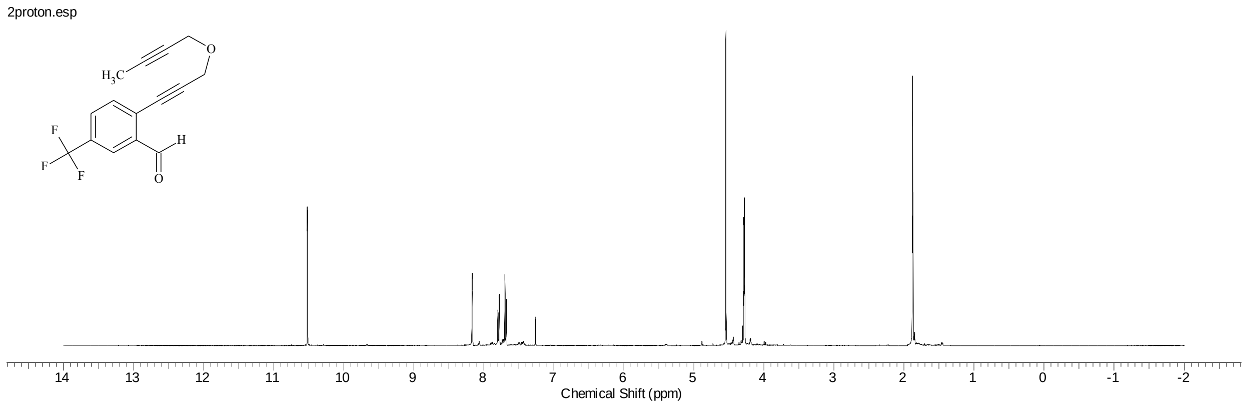

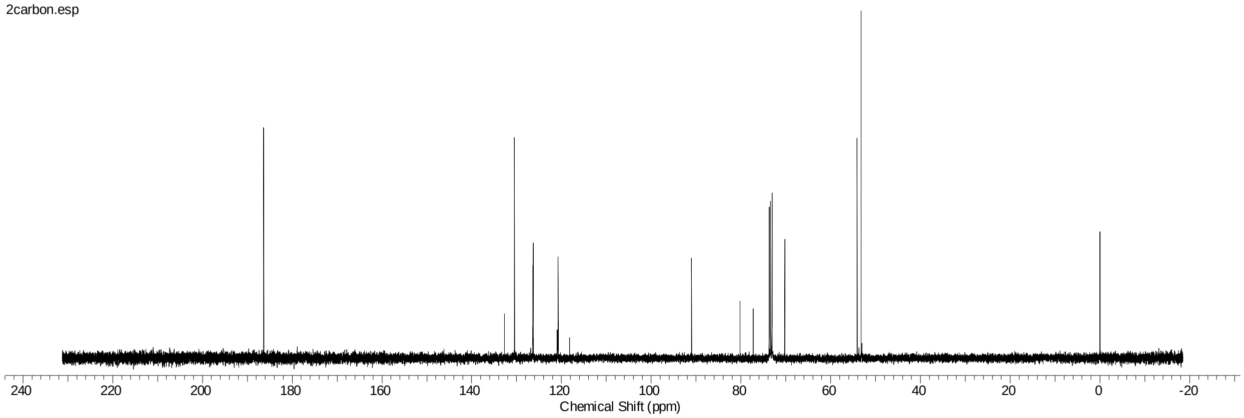

^{1}H and ^{13}C NMR of compound **10** ($CDCl_3$)

4proton.esp

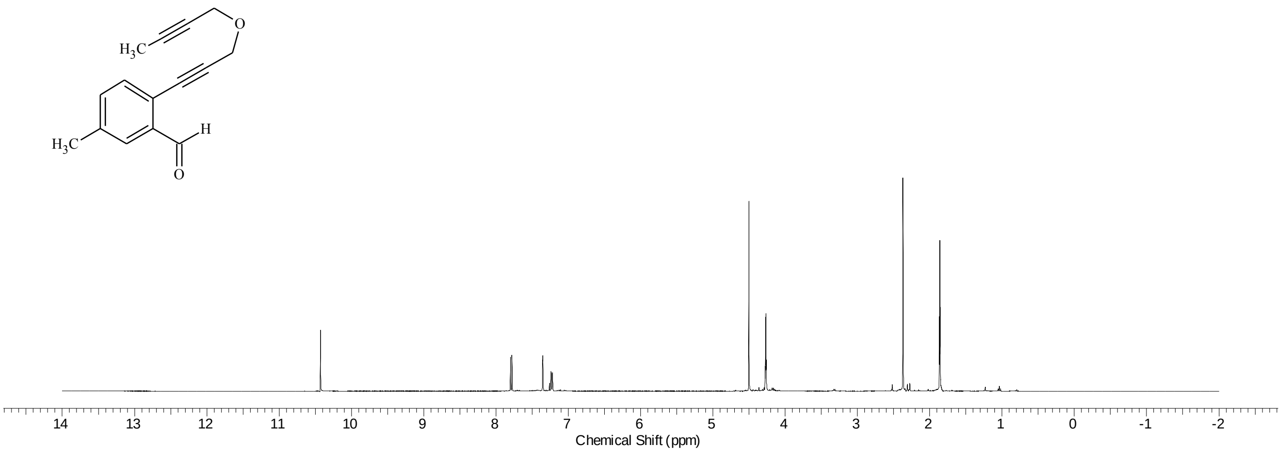

4carbon.esp

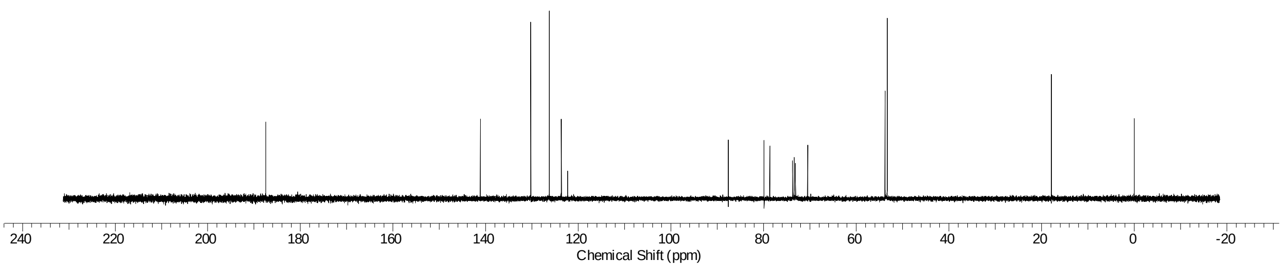

^{1}H and ^{13}C NMR of compound **12** ($CDCl_3$)

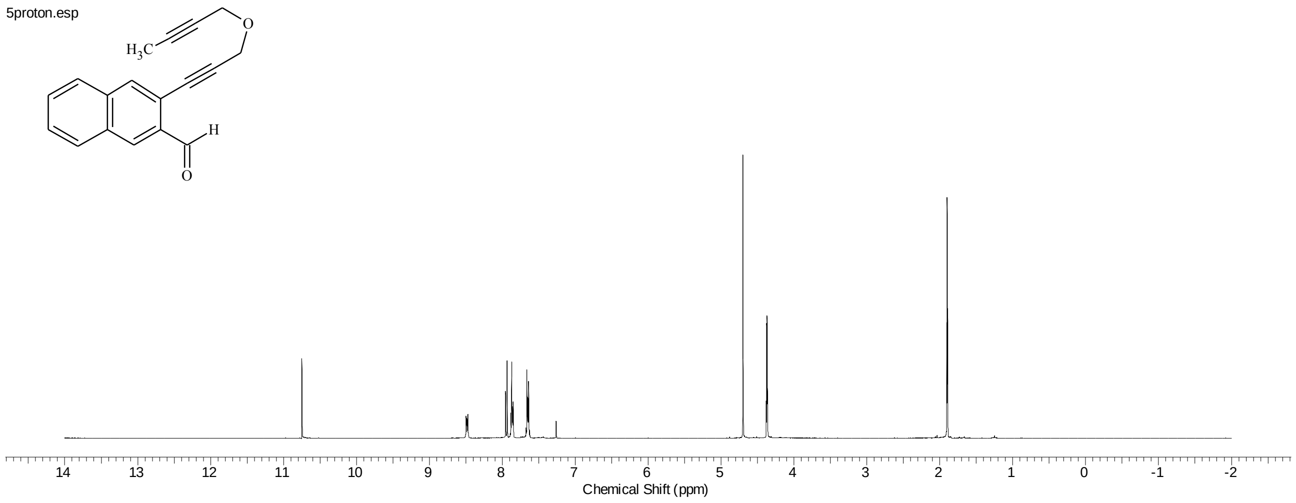

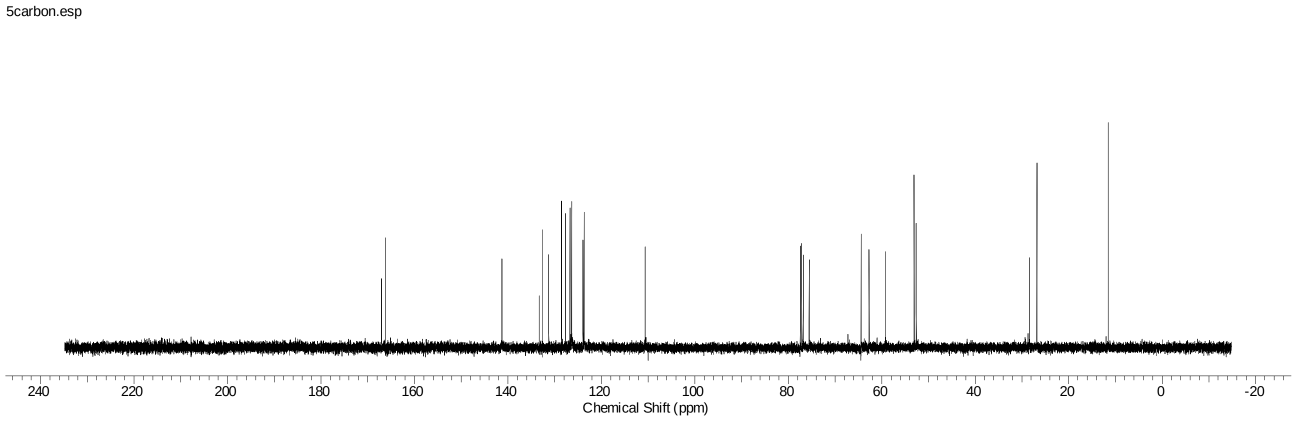

^{1}H and ^{13}C NMR of compound **14** ($CDCl_3$)

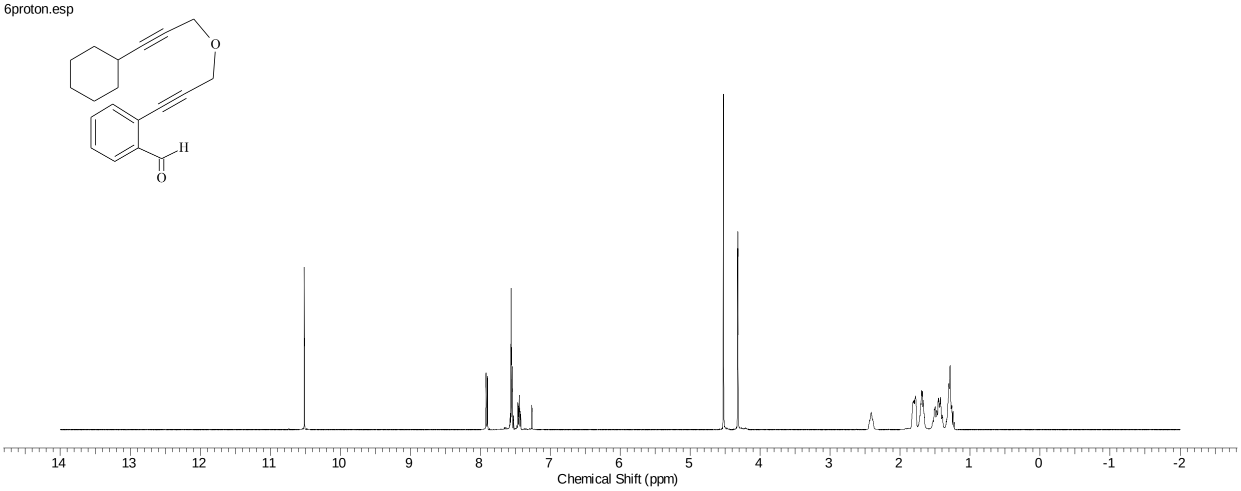

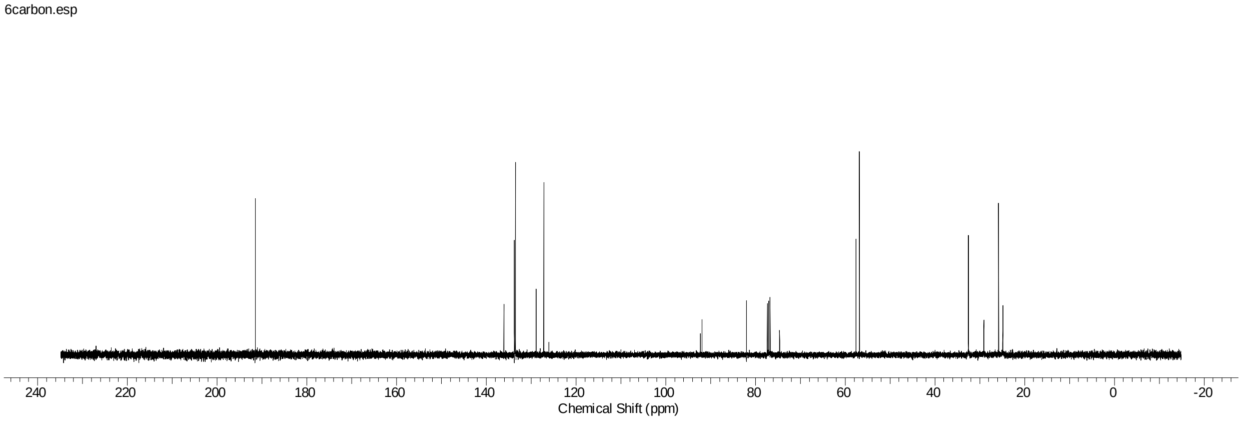

^{1}H and ^{13}C NMR of compound **16** ($CDCl_3$)

7proton.esp

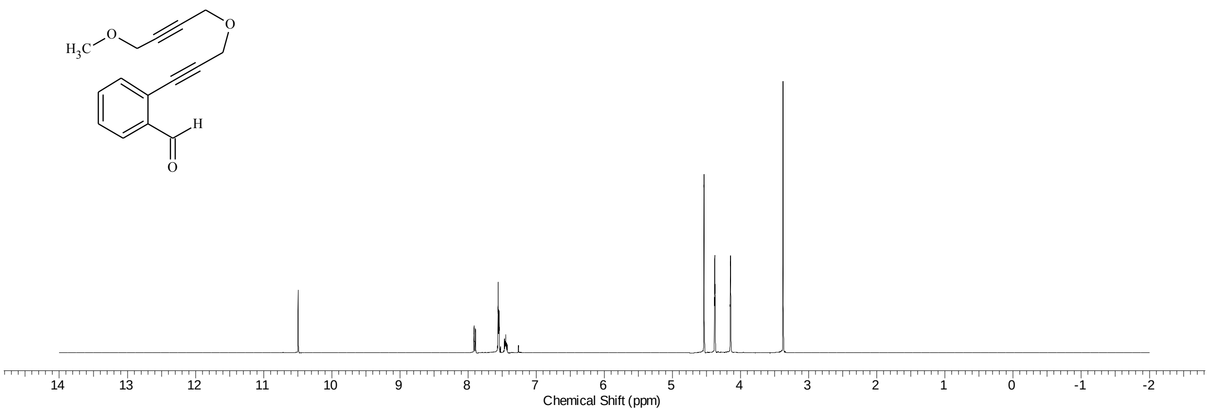

7carbon.esp

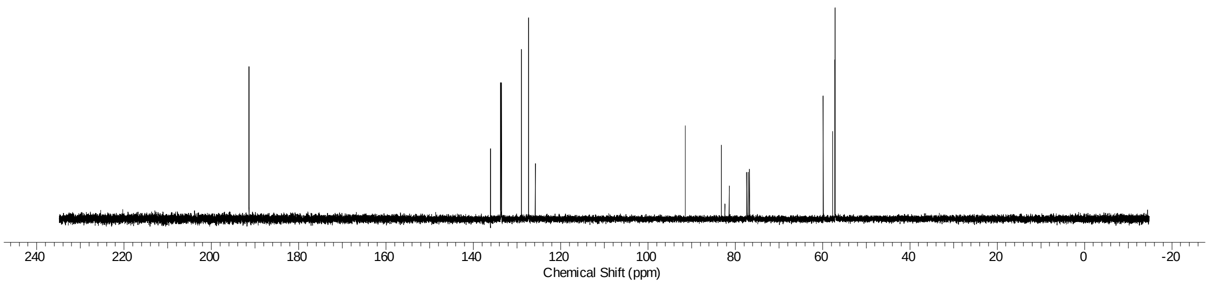

^{1}H and ^{13}C NMR of compound **18** ($CDCl_3$)

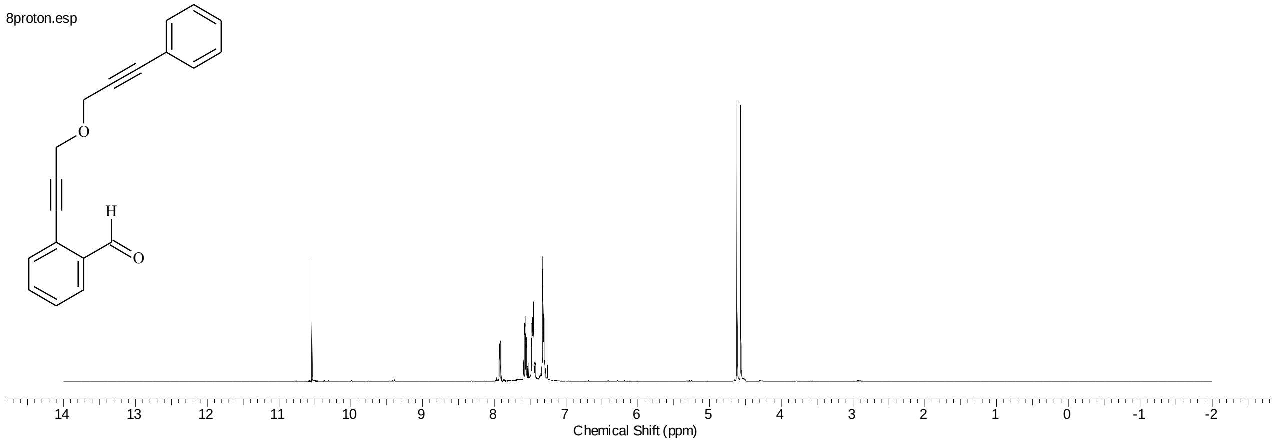

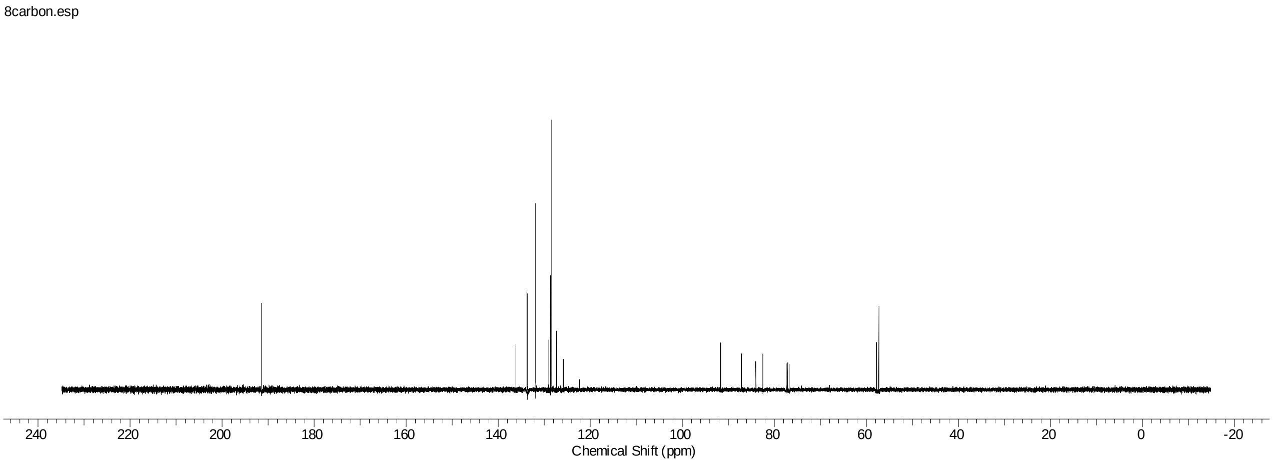

^{1}H and ^{13}C NMR of compound **20** ($CDCl_3$)

10proton.esp

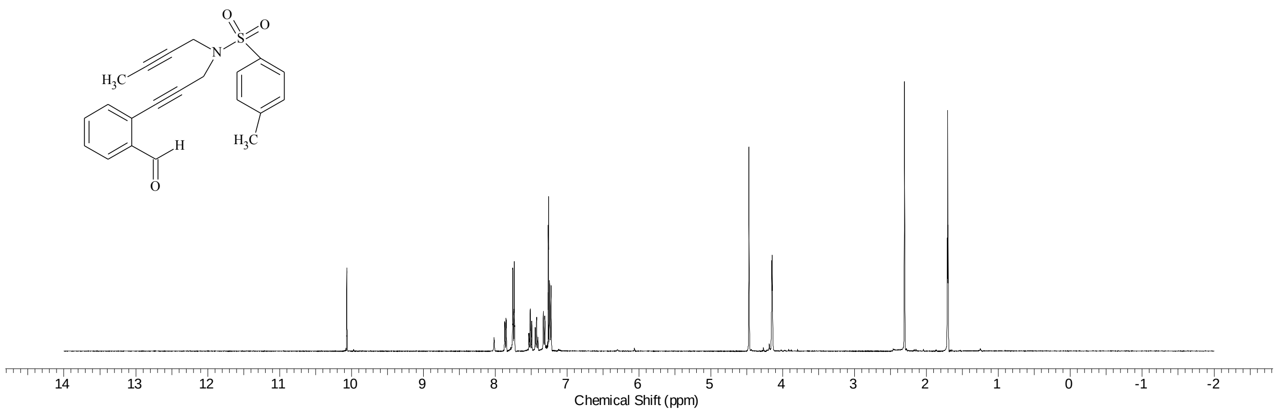

10carbon.esp

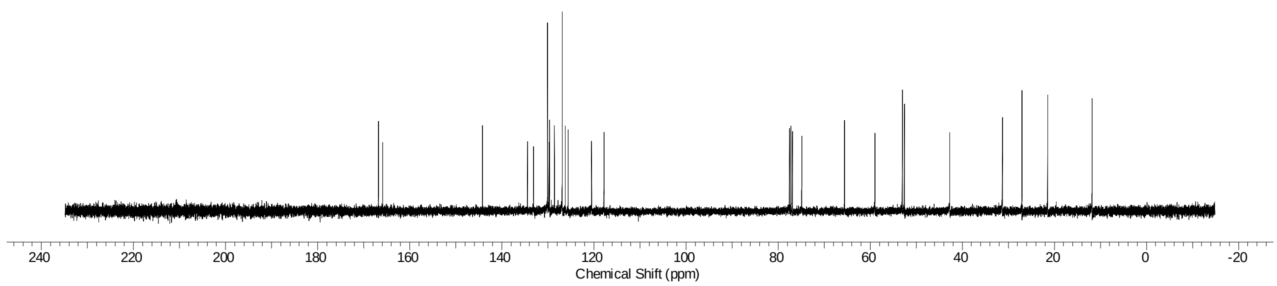

^{1}H and ^{13}C NMR of compound **22** ($CDCl_3$)

11proton.esp

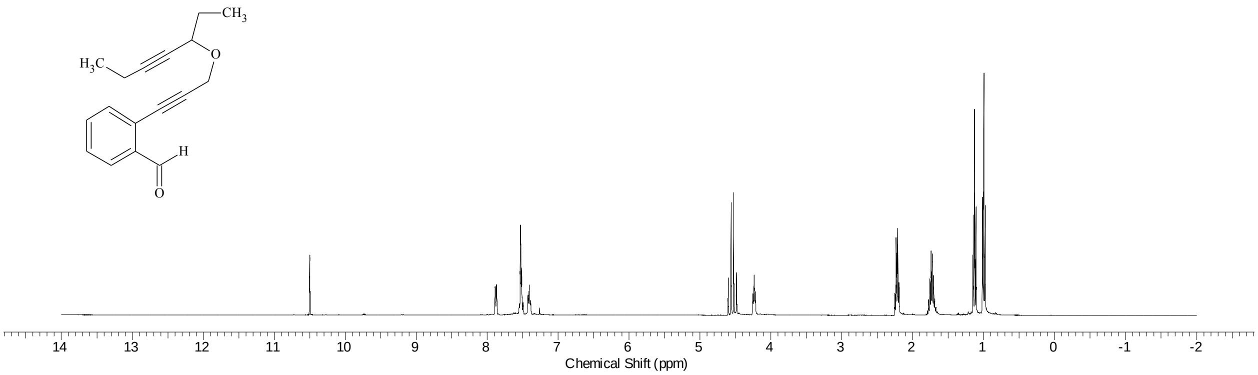

11carbon.esp

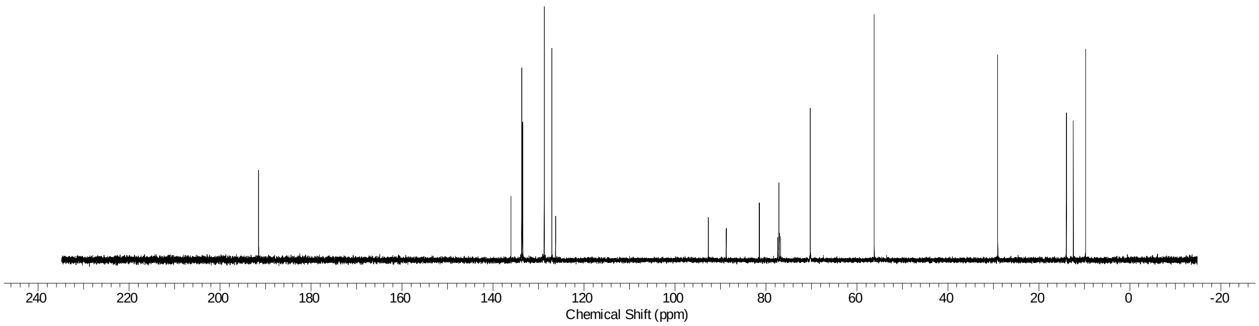

^{1}H and ^{13}C NMR of compound **6** ($CDCl_3$)

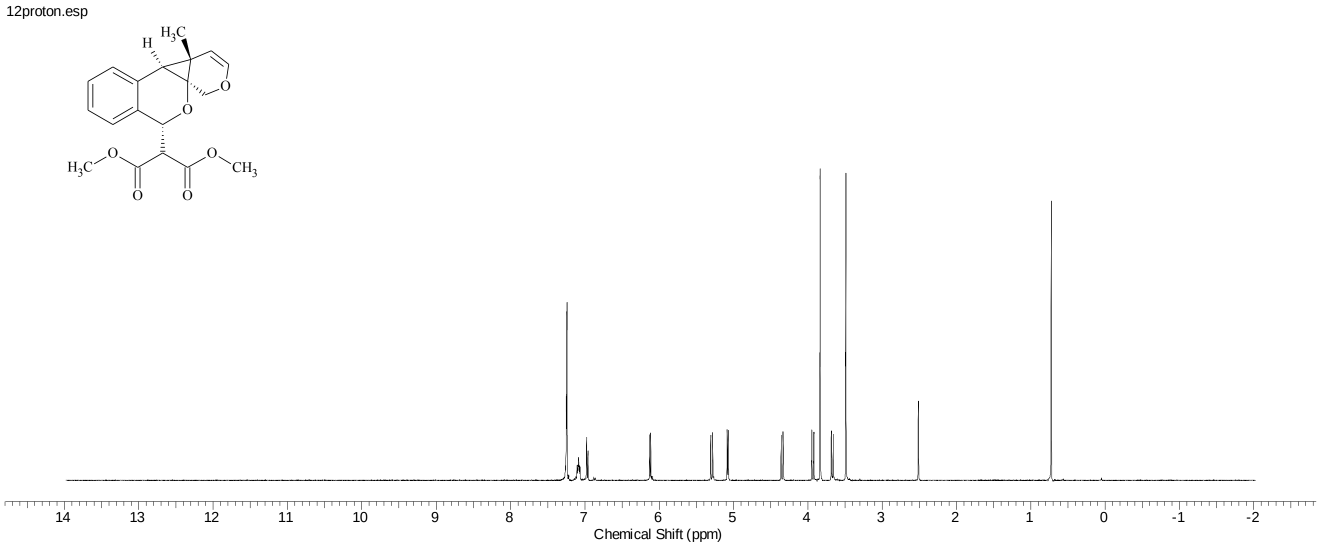

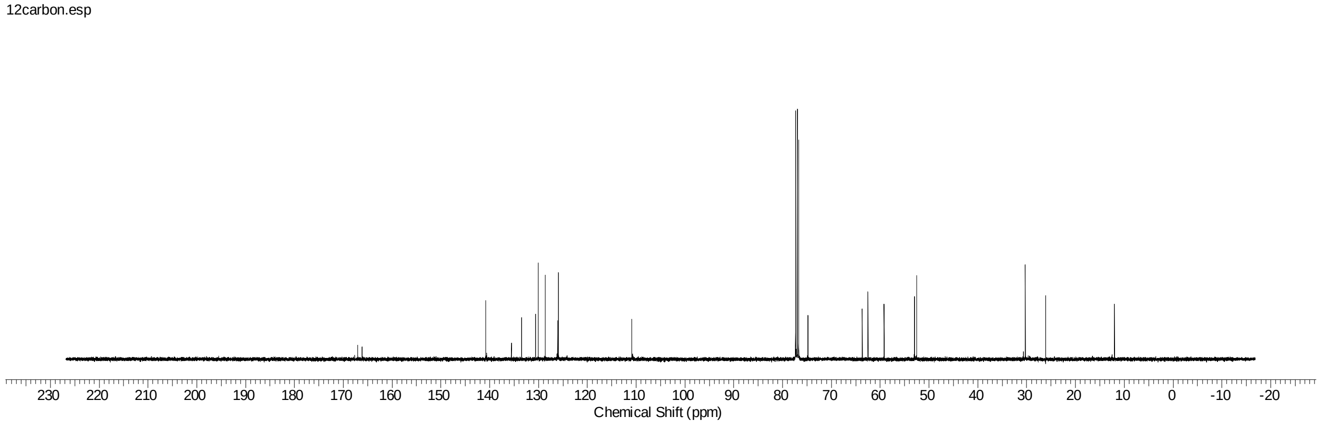

^{1}H and ^{13}C NMR of compound **9** ($CDCl_3$)

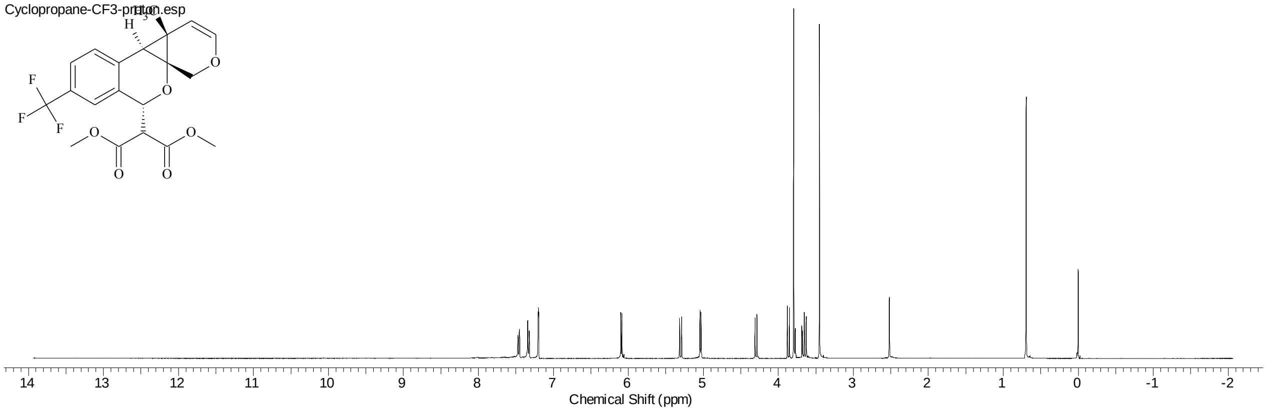

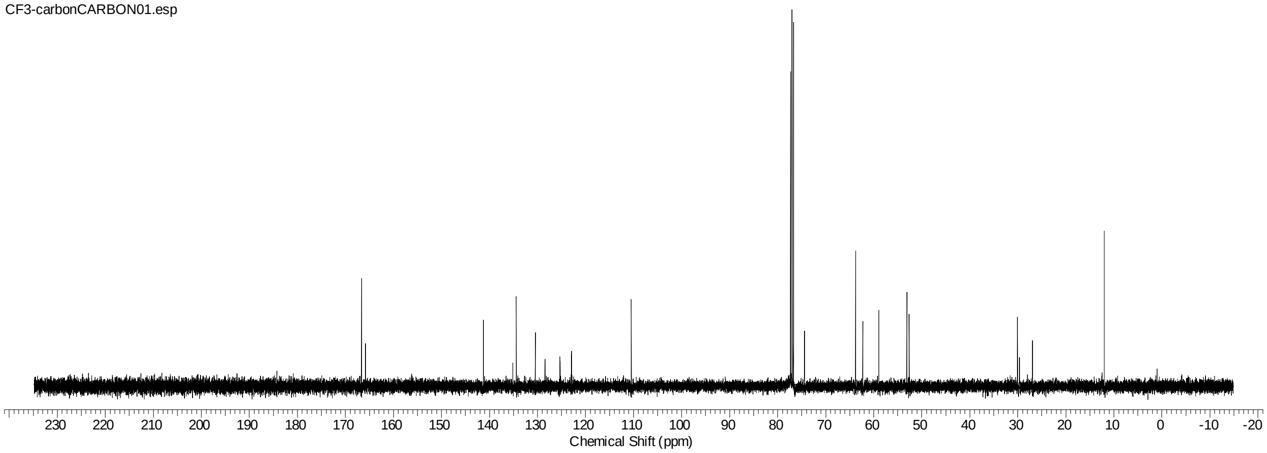

^{1}H and ^{13}C NMR of compound **11** ($CDCl_3$)

14proton.esp

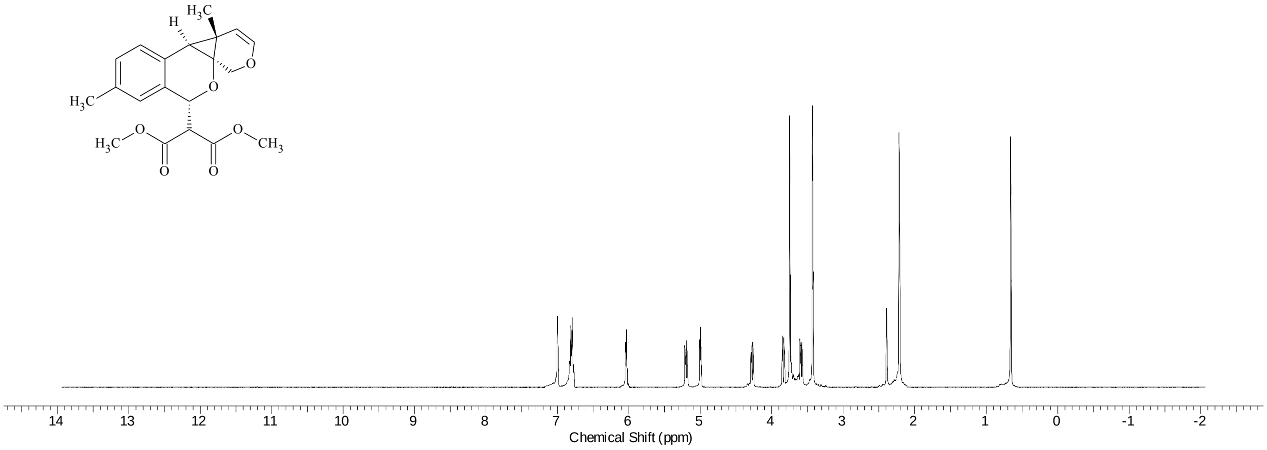

14carbon.esp

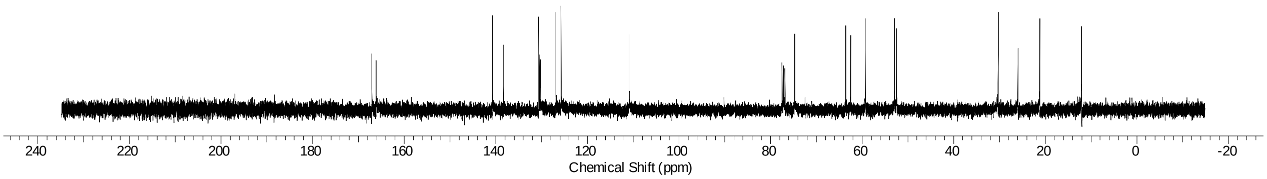

^{1}H and ^{13}C NMR of compound **13** ($CDCl_3$)

15proton.esp

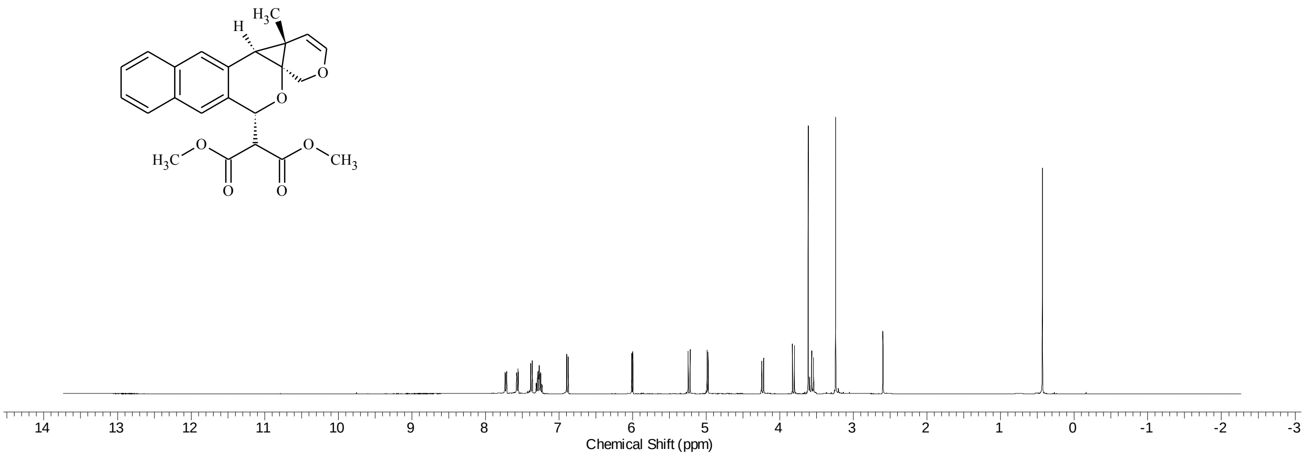

15carbon.esp

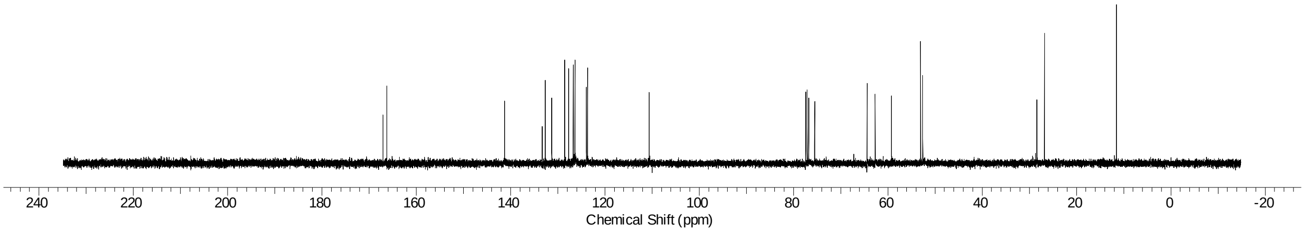

^{1}H and ^{13}C NMR of compound **17** ($CDCl_3$)

17proton.esp

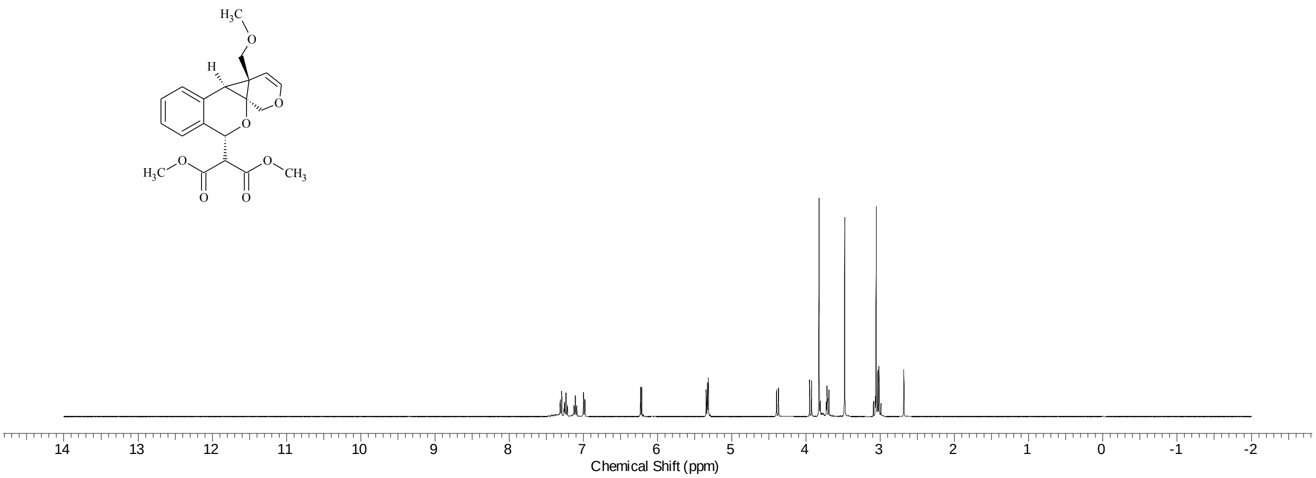

17carbon.esp

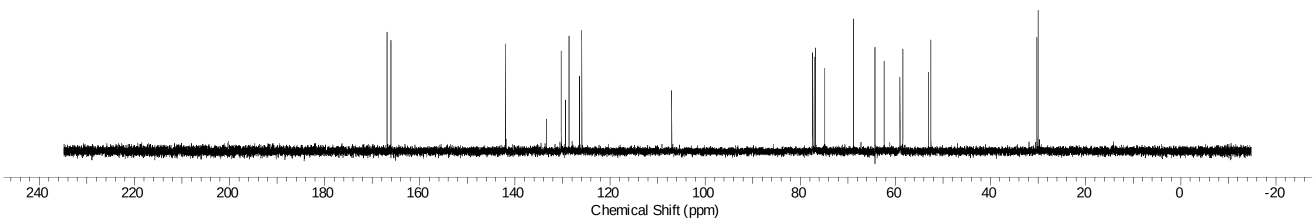

^{1}H and ^{13}C NMR of compound **21** ($CDCl_3$)

19proton.esp

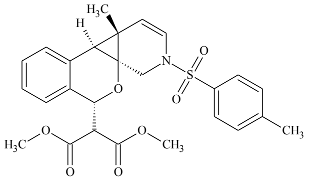

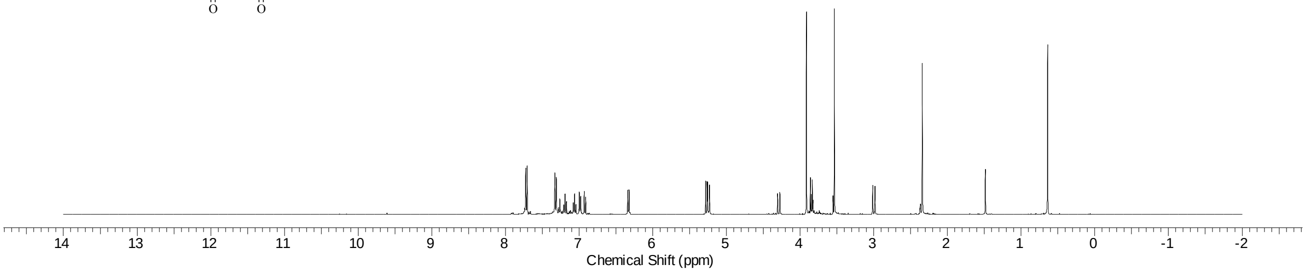

19carbon.esp

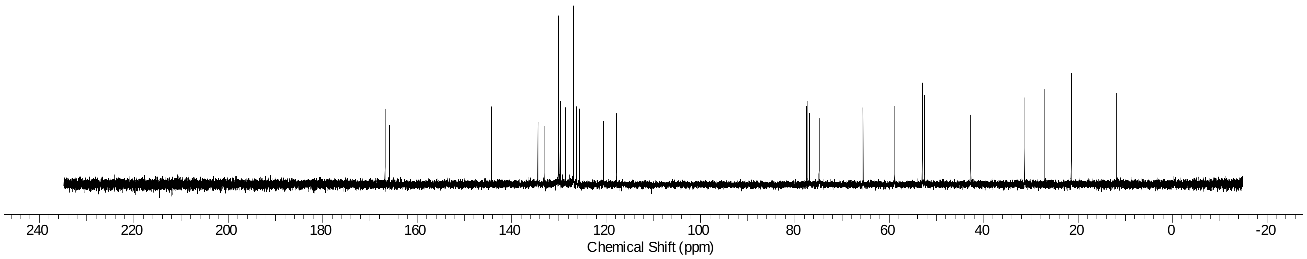

^{1}H and ^{13}C NMR of compound **19** ($CDCl_3$)

Ph-cyclopropanePROTON01

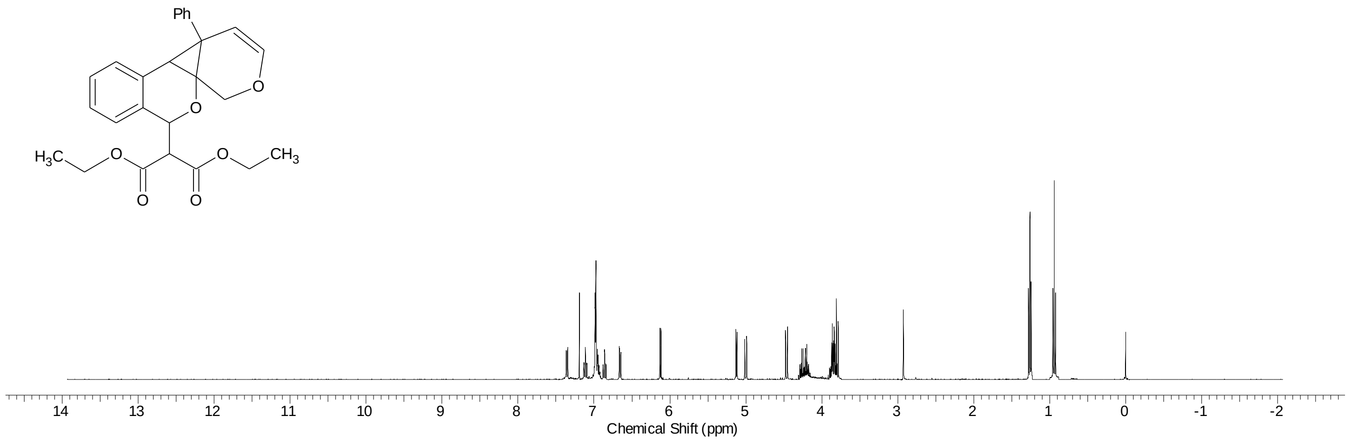

ph-carbon.CARBON01.esp

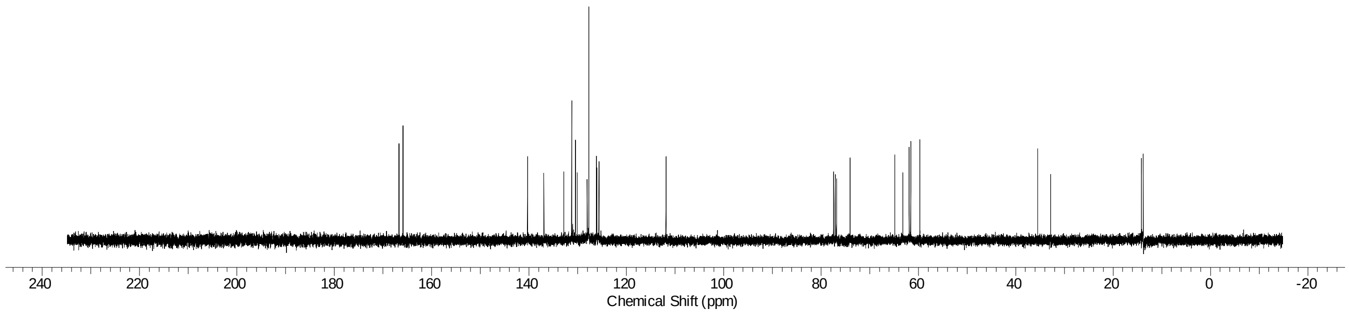

^{1}H and ^{13}C NMR of compound **23** ($CDCl_3$)

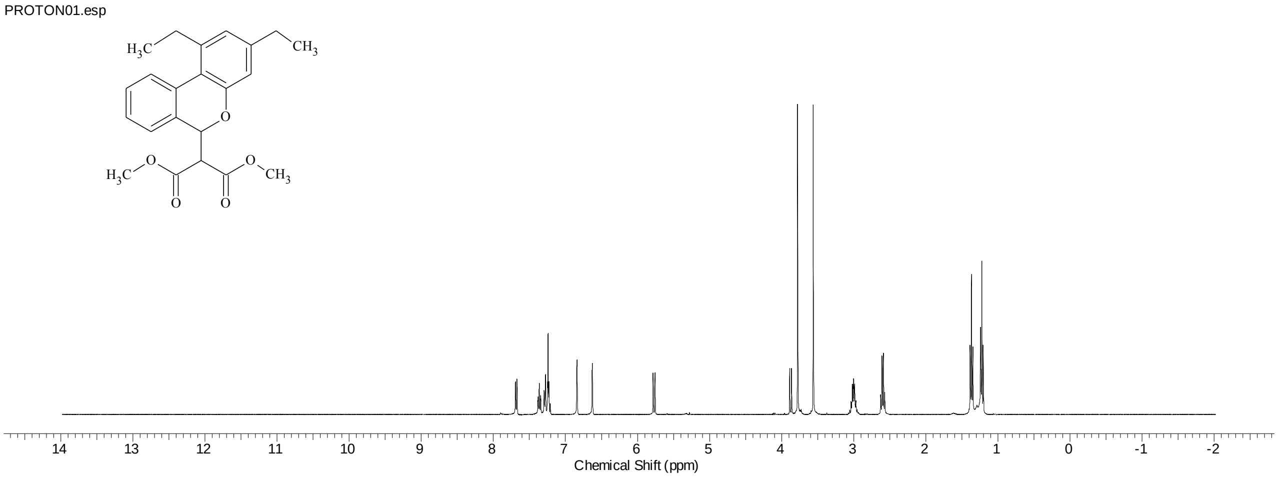

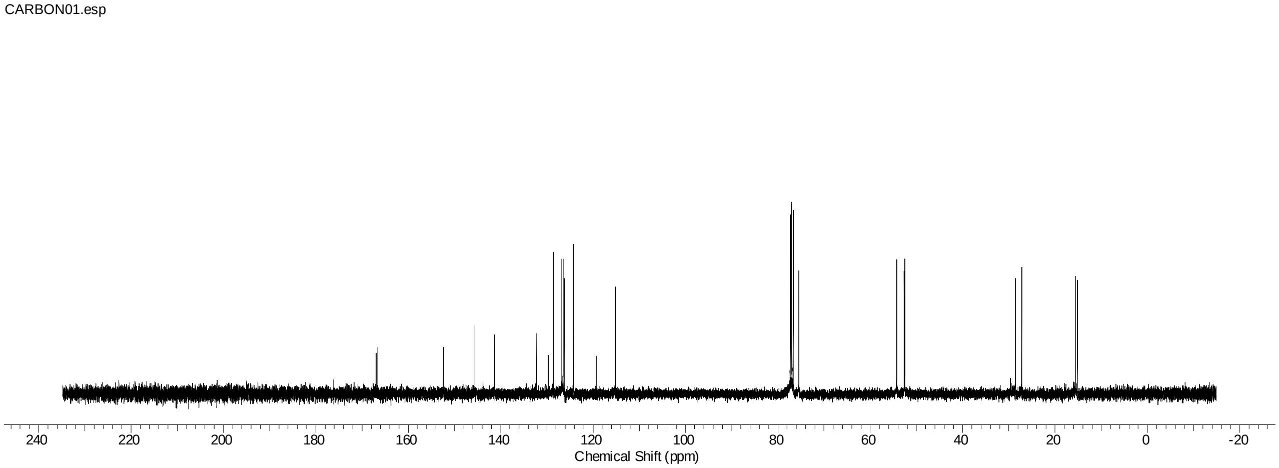

HMBC for compound **23** ($CDCl_3$)

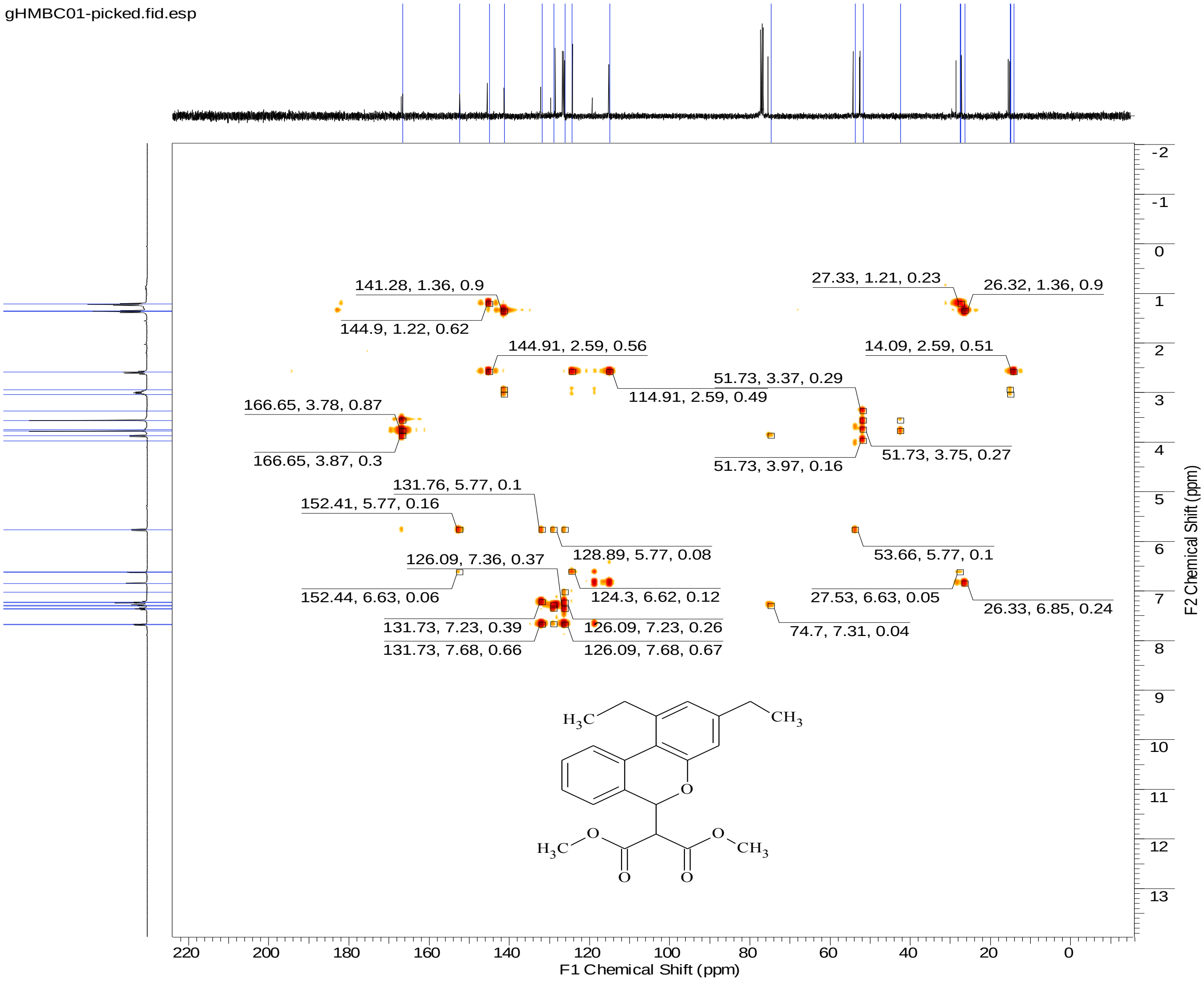

HMQC for compound **23** ($CDCl_3$)

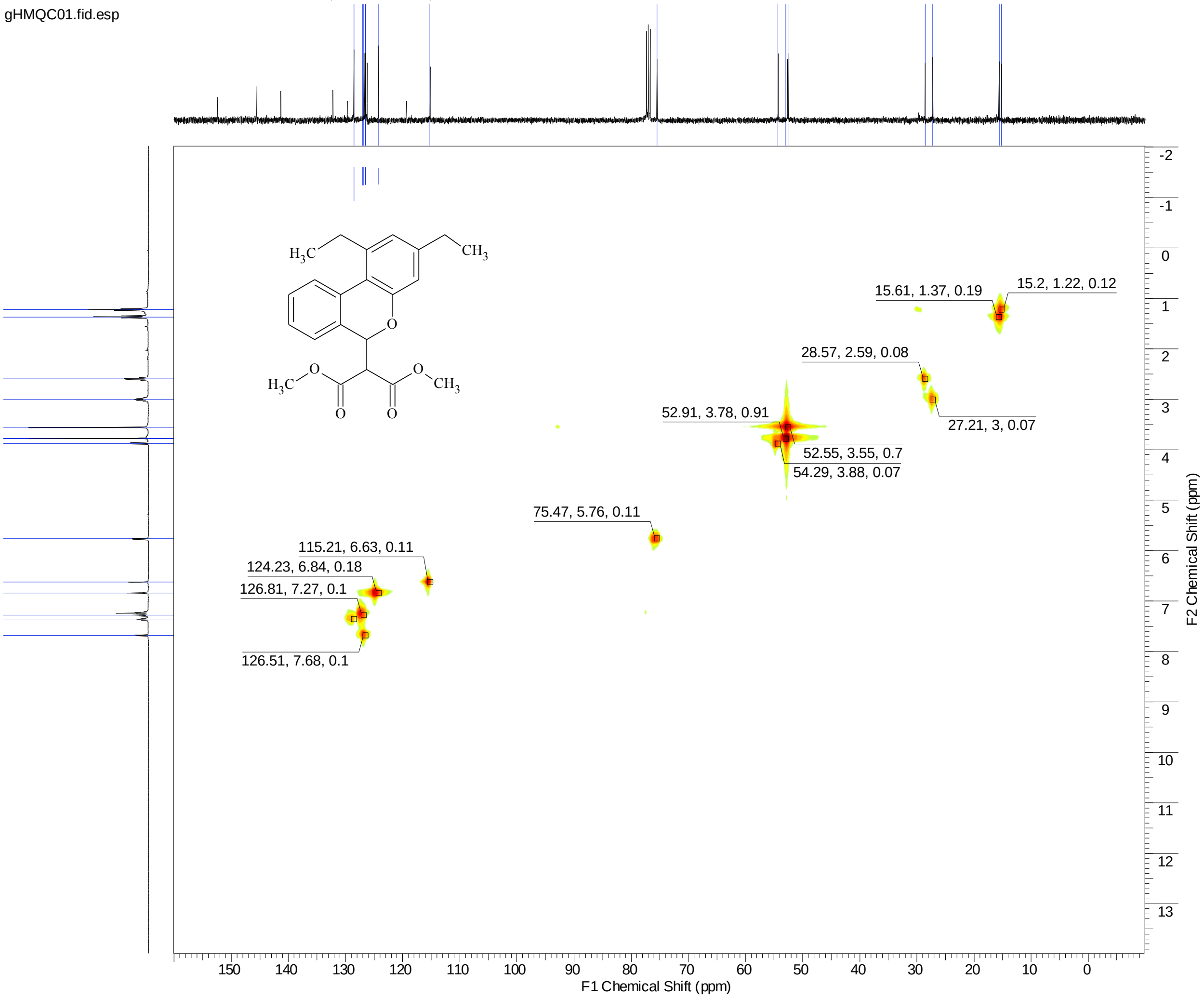

X-ray crystal structure data

Crystals of compound **6** suitable for X-ray diffraction analysis were obtained by slow evaporation from CH_2Cl_2. Crystallographic data have been deposited with the Cambridge Crystallographic Data Centre (CCDC 882923). Copies of the data can be obtained free of charge on application to the CCDC, 12 Union Road, Cambridge CB21EZ, UK (fax: (+44)-1223-336-033; email:deposit@ccdc.cam.ac.uk.)

Table 1. Crystal data and structure refinement for **6**.

Identification code	compound **6**	
Empirical formula	C19 H20 O6	
Formula weight	344.35	
Temperature	173(2) K	
Wavelength	0.71073 Å	
Crystal system	Triclinic	
Space group	P-1	
Unit cell dimensions	a = 9.6011(10) Å	α= 86.442(5)°.
	b = 9.9605(11) Å	β= 83.835(5)°.
	c = 10.0481(9) Å	γ = 61.309(6)°.
Volume	838.00(15) Å^3	
Z	2	
Density (calculated)	1.365 Mg/m^3	
Absorption coefficient	0.102 mm^{-1}	
F(000)	364	
Crystal size	0.40 x 0.30 x 0.20 mm^3	
Theta range for data collection	2.04 to 33.14°.	
Index ranges	-14<=h<=14, -15<=k<=15, -15<=l<=15	
Reflections collected	27145	
Independent reflections	6349 [R(int) = 0.0292]	
Completeness to theta = 33.14°	99.3 %	
Absorption correction	Semi-empirical from equivalents	
Max. and min. transmission	0.9800 and 0.9605	
Refinement method	Full-matrix least-squares on F^2	
Data / restraints / parameters	6349 / 0 / 306	
Goodness-of-fit on F^2	1.122	
Final R indices [I>2sigma(I)]	R1 = 0.0415, wR2 = 0.1248	
R indices (all data)	R1 = 0.0527, wR2 = 0.1318	

Largest diff. peak and hole 0.396 and -0.268 e.Å^{-3}

Table 2. Atomic coordinates (x 10^4) and equivalent isotropic displacement parameters (Å^{2}x 10^3) for **6**. U(eq) is defined as one third of the trace of the orthogonalized U^{ij} tensor.

	x	y	z	U(eq)
O(1)	3401(1)	7609(1)	1989(1)	22(1)
O(2)	3016(1)	10713(1)	-394(1)	30(1)
O(3)	7524(1)	4670(1)	3821(1)	35(1)
O(4)	8593(1)	6182(1)	3137(1)	36(1)
O(5)	6331(1)	4376(1)	1162(1)	33(1)
O(6)	7198(1)	5850(1)	-36(1)	32(1)
C(1)	4586(1)	6952(1)	2927(1)	21(1)
C(2)	4170(1)	7974(1)	4114(1)	20(1)
C(3)	4543(1)	7362(1)	5394(1)	27(1)
C(4)	4158(1)	8316(1)	6474(1)	30(1)
C(5)	3391(1)	9887(1)	6283(1)	27(1)
C(6)	2991(1)	10505(1)	5018(1)	23(1)
C(7)	3372(1)	9559(1)	3922(1)	19(1)
C(8)	2932(1)	10192(1)	2566(1)	19(1)
C(9)	3000(1)	9117(1)	1558(1)	19(1)
C(10)	3504(1)	9184(1)	91(1)	25(1)
C(11)	1444(1)	11733(1)	-48(1)	31(1)
C(12)	632(1)	11595(1)	1056(1)	27(1)
C(13)	1380(1)	10347(1)	2051(1)	21(1)
C(14)	275(1)	9951(1)	2949(1)	28(1)
C(15)	6226(1)	6559(1)	2191(1)	21(1)
C(16)	7586(1)	5807(1)	3095(1)	24(1)
C(17)	8767(2)	3879(2)	4711(2)	50(1)
C(18)	6570(1)	5456(1)	1061(1)	24(1)
C(19)	7557(1)	4917(2)	-1201(1)	38(1)

Table 3. Bond lengths [Å] and angles [°] for **6**.

O(1)-C(9)	1.4115(10)
O(1)-C(1)	1.4318(9)
O(2)-C(11)	1.3747(12)
O(2)-C(10)	1.4304(11)
O(3)-C(16)	1.3303(11)
O(3)-C(17)	1.4438(13)
O(4)-C(16)	1.1977(11)
O(5)-C(18)	1.1997(11)
O(6)-C(18)	1.3280(11)
O(6)-C(19)	1.4465(12)
C(1)-C(2)	1.5087(11)
C(1)-C(15)	1.5400(11)
C(1)-H(1)	0.980(12)
C(2)-C(3)	1.3953(11)
C(2)-C(7)	1.3961(11)
C(3)-C(4)	1.3860(13)
C(3)-H(3)	0.952(12)
C(4)-C(5)	1.3836(15)
C(4)-H(4)	0.952(15)
C(5)-C(6)	1.3859(12)
C(5)-H(5)	0.986(14)
C(6)-C(7)	1.3960(11)
C(6)-H(6)	0.992(13)
C(7)-C(8)	1.4801(10)
C(8)-C(9)	1.4935(11)
C(8)-C(13)	1.5636(11)
C(8)-H(8)	0.978(12)
C(9)-C(13)	1.4985(11)
C(9)-C(10)	1.5089(11)
C(10)-H(10B)	1.000(15)
C(10)-H(10A)	0.990(12)
C(11)-C(12)	1.3249(13)
C(11)-H(11)	0.948(15)
C(12)-C(13)	1.4847(12)

C(12)-H(12)	0.961(15)
C(13)-C(14)	1.4996(11)
C(14)-H(14C)	0.969(15)
C(14)-H(14B)	0.999(15)
C(14)-H(14A)	0.975(17)
C(15)-C(16)	1.5239(11)
C(15)-C(18)	1.5266(11)
C(15)-H(15)	0.965(11)
C(17)-H(17B)	0.982(19)
C(17)-H(17A)	0.922(17)
C(17)-H(17C)	0.932(19)
C(19)-H(19C)	0.943(18)
C(19)-H(19B)	0.975(18)
C(19)-H(19A)	0.972(19)

C(9)-O(1)-C(1)	115.65(6)
C(11)-O(2)-C(10)	114.56(7)
C(16)-O(3)-C(17)	115.83(8)
C(18)-O(6)-C(19)	116.19(8)
O(1)-C(1)-C(2)	111.78(6)
O(1)-C(1)-C(15)	109.00(6)
C(2)-C(1)-C(15)	113.00(6)
O(1)-C(1)-H(1)	103.4(8)
C(2)-C(1)-H(1)	110.1(7)
C(15)-C(1)-H(1)	109.1(7)
C(3)-C(2)-C(7)	119.86(7)
C(3)-C(2)-C(1)	121.17(7)
C(7)-C(2)-C(1)	118.96(7)
C(4)-C(3)-C(2)	120.47(8)
C(4)-C(3)-H(3)	120.6(8)
C(2)-C(3)-H(3)	118.9(8)
C(5)-C(4)-C(3)	119.80(8)
C(5)-C(4)-H(4)	122.2(9)
C(3)-C(4)-H(4)	118.0(9)
C(4)-C(5)-C(6)	120.11(8)
C(4)-C(5)-H(5)	123.6(8)

C(6)-C(5)-H(5)	116.3(8)
C(5)-C(6)-C(7)	120.73(8)
C(5)-C(6)-H(6)	120.5(8)
C(7)-C(6)-H(6)	118.7(8)
C(6)-C(7)-C(2)	119.00(7)
C(6)-C(7)-C(8)	121.74(7)
C(2)-C(7)-C(8)	119.25(7)
C(7)-C(8)-C(9)	116.97(6)
C(7)-C(8)-C(13)	120.01(6)
C(9)-C(8)-C(13)	58.65(5)
C(7)-C(8)-H(8)	114.1(7)
C(9)-C(8)-H(8)	118.7(7)
C(13)-C(8)-H(8)	117.6(7)
O(1)-C(9)-C(8)	117.55(6)
O(1)-C(9)-C(13)	116.45(6)
C(8)-C(9)-C(13)	63.01(5)
O(1)-C(9)-C(10)	111.73(6)
C(8)-C(9)-C(10)	123.34(7)
C(13)-C(9)-C(10)	116.41(6)
O(2)-C(10)-C(9)	112.85(7)
O(2)-C(10)-H(10B)	109.3(8)
C(9)-C(10)-H(10B)	107.3(8)
O(2)-C(10)-H(10A)	105.1(7)
C(9)-C(10)-H(10A)	111.6(7)
H(10B)-C(10)-H(10A)	110.7(10)
C(12)-C(11)-O(2)	123.28(8)
C(12)-C(11)-H(11)	125.3(9)
O(2)-C(11)-H(11)	111.4(9)
C(11)-C(12)-C(13)	122.11(8)
C(11)-C(12)-H(12)	119.5(9)
C(13)-C(12)-H(12)	118.3(9)
C(12)-C(13)-C(9)	113.42(7)
C(12)-C(13)-C(14)	116.38(7)
C(9)-C(13)-C(14)	120.29(7)
C(12)-C(13)-C(8)	115.21(7)
C(9)-C(13)-C(8)	58.34(5)

C(14)-C(13)-C(8)	120.57(7)
C(13)-C(14)-H(14C)	112.0(8)
C(13)-C(14)-H(14B)	110.4(9)
H(14C)-C(14)-H(14B)	110.9(12)
C(13)-C(14)-H(14A)	110.1(9)
H(14C)-C(14)-H(14A)	105.4(13)
H(14B)-C(14)-H(14A)	107.8(14)
C(16)-C(15)-C(18)	108.06(6)
C(16)-C(15)-C(1)	113.27(7)
C(18)-C(15)-C(1)	108.80(6)
C(16)-C(15)-H(15)	106.7(7)
C(18)-C(15)-H(15)	111.5(7)
C(1)-C(15)-H(15)	108.5(7)
O(4)-C(16)-O(3)	124.22(8)
O(4)-C(16)-C(15)	124.26(8)
O(3)-C(16)-C(15)	111.51(7)
O(3)-C(17)-H(17B)	111.1(11)
O(3)-C(17)-H(17A)	111.1(11)
H(17B)-C(17)-H(17A)	106.2(15)
O(3)-C(17)-H(17C)	102.6(12)
H(17B)-C(17)-H(17C)	115.3(16)
H(17A)-C(17)-H(17C)	110.7(15)
O(5)-C(18)-O(6)	125.15(8)
O(5)-C(18)-C(15)	124.66(8)
O(6)-C(18)-C(15)	110.18(7)
O(6)-C(19)-H(19C)	108.8(11)
O(6)-C(19)-H(19B)	106.7(10)
H(19C)-C(19)-H(19B)	114.6(15)
O(6)-C(19)-H(19A)	105.2(11)
H(19C)-C(19)-H(19A)	110.1(15)
H(19B)-C(19)-H(19A)	111.0(15)

Symmetry transformations used to generate equivalent atoms:

Table 4. Anisotropic displacement parameters ($Å^2$x 10^3) for **6**. The anisotropic displacement factor exponent takes the form: $-2\pi^2[h^2 a^{*2}U^{11} + ... + 2 h k a^* b^* U^{12}]$

	U^{11}	U^{22}	U^{33}	U^{23}	U^{13}	U^{12}
O(1)	22(1)	19(1)	24(1)	-1(1)	-6(1)	-9(1)
O(2)	27(1)	36(1)	25(1)	9(1)	-2(1)	-13(1)
O(3)	34(1)	29(1)	45(1)	12(1)	-18(1)	-16(1)
O(4)	31(1)	41(1)	43(1)	6(1)	-12(1)	-21(1)
O(5)	38(1)	24(1)	37(1)	-4(1)	-3(1)	-15(1)
O(6)	34(1)	37(1)	29(1)	-10(1)	5(1)	-20(1)
C(1)	21(1)	18(1)	22(1)	1(1)	-3(1)	-9(1)
C(2)	21(1)	22(1)	19(1)	1(1)	-2(1)	-11(1)
C(3)	30(1)	29(1)	22(1)	6(1)	-4(1)	-14(1)
C(4)	31(1)	43(1)	18(1)	3(1)	-2(1)	-20(1)
C(5)	24(1)	41(1)	20(1)	-7(1)	2(1)	-18(1)
C(6)	20(1)	27(1)	23(1)	-6(1)	1(1)	-12(1)
C(7)	17(1)	22(1)	18(1)	-1(1)	0(1)	-10(1)
C(8)	19(1)	19(1)	20(1)	0(1)	-2(1)	-9(1)
C(9)	17(1)	20(1)	18(1)	0(1)	-2(1)	-8(1)
C(10)	23(1)	30(1)	19(1)	-1(1)	-1(1)	-10(1)
C(11)	27(1)	32(1)	30(1)	10(1)	-9(1)	-12(1)
C(12)	20(1)	25(1)	30(1)	4(1)	-5(1)	-6(1)
C(13)	17(1)	22(1)	21(1)	1(1)	-2(1)	-8(1)
C(14)	21(1)	33(1)	30(1)	1(1)	2(1)	-13(1)
C(15)	20(1)	18(1)	23(1)	-1(1)	-3(1)	-8(1)
C(16)	22(1)	22(1)	27(1)	-2(1)	-4(1)	-8(1)
C(17)	47(1)	42(1)	62(1)	23(1)	-32(1)	-20(1)
C(18)	18(1)	22(1)	28(1)	-3(1)	-4(1)	-6(1)
C(19)	34(1)	49(1)	31(1)	-16(1)	4(1)	-19(1)

Table 5. Hydrogen coordinates (x 10^4) and isotropic displacement parameters ($Å^2$x 10^{3}) for **6**.

	x	y	z	U(eq)
H(1)	4569(15)	6001(14)	3213(13)	30(3)
H(3)	5071(15)	6279(14)	5512(13)	30(3)
H(4)	4428(17)	7855(16)	7332(15)	42(3)
H(5)	3063(17)	10625(16)	7013(15)	42(4)
H(6)	2395(15)	11630(15)	4882(13)	33(3)
H(8)	3252(14)	10966(13)	2270(12)	26(3)
H(10B)	3008(17)	8712(16)	-413(15)	41(3)
H(10A)	4679(14)	8640(13)	-92(12)	25(3)
H(11)	1029(17)	12520(16)	-703(15)	42(4)
H(12)	-465(18)	12346(16)	1244(14)	41(3)
H(14C)	820(17)	9222(16)	3644(15)	42(4)
H(14B)	-644(19)	10897(17)	3344(15)	48(4)
H(14A)	-151(19)	9451(18)	2438(17)	50(4)
H(15)	6205(14)	7496(13)	1853(12)	24(3)
H(17B)	8830(20)	4600(20)	5297(19)	64(5)
H(17A)	9750(20)	3382(18)	4237(17)	52(4)
H(17C)	8490(20)	3180(20)	5150(20)	71(5)
H(19C)	8140(20)	3880(20)	-952(18)	64(5)
H(19B)	6540(20)	5193(19)	-1546(17)	60(5)
H(19A)	8210(20)	5210(20)	-1831(19)	66(5)
